# A Suitable Membrane Distance Regulated by the RBD_ACE2 Interaction is Critical for SARS‐CoV‐2 Spike‐Mediated Viral Invasion

**DOI:** 10.1002/advs.202301478

**Published:** 2023-08-17

**Authors:** Mengdan Wu, Wei Li, Sheng Lin, Jiaqi Fan, Lele Cui, Yijuan Xiang, Kaiyu Li, Linwei Tang, Yanping Duan, Zimin Chen, Fanli Yang, Weiwei Shui, Guangwen Lu, Ying Lai

**Affiliations:** ^1^ National Clinical Research Center for Geriatrics State Key Laboratory of Biotherapy West China Hospital Sichuan University Chengdu Sichuan 610041 China; ^2^ West China Hospital Emergency Department State Key Laboratory of Biotherapy West China Hospital Sichuan University Chengdu Sichuan 610041 China

**Keywords:** membrane fusion, RBD_ACE2 interaction, SARS‐CoV‐2, spike

## Abstract

The receptor‐binding domain (RBD) of spike recognizing the receptor angiotensin‐converting enzyme 2 (ACE2) initiates membrane fusion between severe acute respiratory syndrome coronavirus 2 (SARS‐CoV‐2) and cell membrane. Although the structure of the RBD_ACE2 complex has been well studied, its functional mechanism in membrane fusion is still not fully understood. Here, using an in vitro cell–vesicle content‐mixing assay, it is found that the cleavage at the S2’ site by thrombin (Thr) protease strongly accelerates membrane fusion, compared to that of cleavage at the S1/S2 site by PreScission (3C) protease. Moreover, mutations at the RBD_ACE2 interface resulted in a positive correlation between binding affinity and fusion probability. In both the cell–vesicle and cell–cell fusion assays, by crosslinking two membranes via the neutravidin (NTV)_biotin interaction or complementary DNA strands, it is found that spike drives membrane fusion in the absence of ACE2, and a suitable distance between two membranes is critical for spike‐mediated membrane fusion. Finally, unsuitable membrane crosslinkers significantly inhibited the fusion probability in the presence of ACE2. Taken together, the results suggest that the RBD_ACE2 complex may act as a crosslinker to bridge the viral and cell membranes at a suitable distance, which is critical, but also substitutable for spike‐mediated SARS‐CoV‐2 entry.

## Graphical abstract

The RBD_ACE2 interaction may function as a membrane crosslinker to bridge the viral and host cell membranes. In in vitro reconstituted system, the RBD_ACE2 interaction is dispensable by a membrane crosslinker that is capable of providing a suitable membrane distance, and spike mediated membrane fusion could be significantly inhibited by unsuitable membrane crosslinkers.

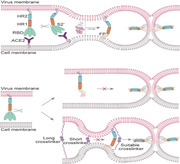

## 1 Introduction

Severe acute respiratory syndrome coronavirus 2 (SARS‐CoV‐2) is continuing to drive the ongoing global coronavirus disease 2019 pandemic. The entry steps of SARS‐CoV‐2 viral particles, which include membrane association and fusion with the host cell membrane,^[^ ^1^, ^2^, ^3^, ^4^ ^]^ are important determinants of viral infections and pathogenesis.^[^ ^1^ ^]^ Similar to other highly transmissible human coronaviruses, such as SARS‐CoV and Middle East respiratory syndrome coronavirus, SARS‐CoV‐2 shares a conserved mechanism of viral entry,^[^ ^5^, ^6^ ^]^ in which spike on the viral membrane docks onto the target cell by S1 subunit recognizing the receptor on the host cell surface, and drives viral membrane fusion with the target cell by S2 subunit, although the viral particle may suffer membrane internalization and endosome acidification in the endocytic pathway.^[^ ^7^, ^8^, ^9^, ^10^, ^11^ ^]^

The spike glycoprotein, located on the viral membrane with a single transmembrane helical region, is a class I fusion protein containing an N‐terminal S1 subunit and a C‐terminal S2 subunit.^[^ ^3^, ^12^ ^]^ In the pre‐fusion state, the N terminal domain and the receptor‐binding domain (RBD) in the S1 subunit weakly associate with helical region 1(HR1) in the S2 subunit to prevent the formation of a six‐helix bundle with HR2.^[^ ^13^ ^]^ Simultaneously, the RBD recognizes the dimer of angiotensin‐converting enzyme 2 (ACE2) on the host cell and initiates membrane fusion by priming the S1/S2 site cleaved by the host protease furin and the S2’ site cleaved by the transmembrane protease serine 2 (TMPRSS2), which results in the disassociation of S1 subunit and the exposure of fusion peptide (FP) to the cell surface.^[^ ^1^, ^13^, ^14^, ^15^, ^16^, ^17^ ^]^ In the S2 subunit, the FP is inserted into the host cell membrane after protease cleavage, and membrane fusion occurs by utilizing the energy produced by the formation of a six‐helix bundle from HR1 and HR2 in a trimeric conformation (i.e., post‐fusion state).^[^ ^12^, ^18^ ^]^

Intensive structural studies have revealed different conformations of spike induced by the interaction with ACE2 via an interface composed of polar contacts formed by the hydrophilic residues,^[^ ^15^ ^]^ indicating that the conformational transition of spike from the pre‐fusion state to the post‐fusion state is critical for viral membrane fusion.^[^ ^1^, ^12^, ^15^, ^19^, ^20^, ^21^ ^]^ Numerous spike variants appear during evolution, and the mutants that alter the binding affinity between RBD and ACE2 also change the efficiency of SARS‐CoV‐2 infection.^[^ ^22^, ^23^, ^24^, ^25^, ^26^, ^27^, ^28^ ^]^ This suggests that the interaction between RBD and ACE2 plays a pivotal role in spike‐dependent viral entry. Notably, one tyrosine‐protein kinase receptor, UFO (AXL), has also been identified as a potential candidate for RBD recognition in the pulmonary and bronchial epithelial cells because its expression level correlates well with SARS‐CoV‐2 infection efficiency.^[^ ^29^ ^]^ Alternatively, SARS‐CoV‐2 can infect the organs with very low ACE2.^[^ ^26^, ^30^, ^31^ ^]^ For instance, glucose‐regulated protein 78 in endothelial cells, which is predominantly expressed on the cell surface and capable of interacting with the RBD, has also been proposed as an alternative accessory factor for SARS‐CoV‐2 based on in silico analysis.^[^ ^30^ ^]^ These results indicate a less conserved function of the RBD_ACE2 interaction in spike‐mediated SARS‐CoV‐2 entry.

In this study, we designed an engineered spike to prevent auto‐cleavage by endogenous proteases during expression, in which the native S1/S2 and S2’ cleavage sites were replaced with PreScission (3C) and thrombin (Thr) cleavage sites, respectively. We then expressed this engineered spike on the surface of human embryonic kidney 293T (HEK293T) cells to mimic the viral membrane and reconstituted the soluble ecto‐domain of ACE2 onto a group of liposomes to mimic the host cell membrane. Using this cell–vesicle fusion assay, we found that membrane fusion efficiency primed by the cleavage at the S2’ site is higher than that by the cleavage at the S1/S2 site. Additionally, mutations that altered the binding affinity of the RBD to ACE2 were positively correlated with their fusion efficiency. Moreover, upon deliberately crosslinking the two membranes, spike was capable of driving membrane fusion even in the absence of ACE2 in both cell–vesicle and cell–cell fusion assays. Furthermore, by crosslinking the two membranes using a pair of DNA strands of different lengths, we found that the optimal distance between the two membranes is critical for spike‐mediated membrane fusion. Finally, the RBD_ACE2 interaction‐initiated membrane fusion was inhibited by manipulating the distance between the two membranes, indicating an alternative strategy for preventing spike‐mediated membrane fusion.

## 2 Results

### 2.1 In Vitro Reconstitution of Spike‐Mediated Membrane Fusion

**Figure 1:**
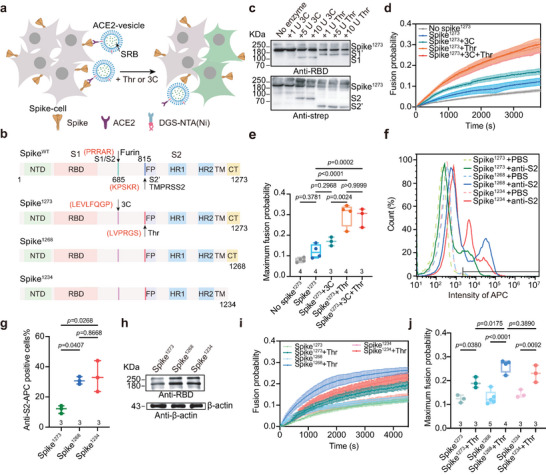
Reconstitution of spike‐mediated viral invasion in the cell–vesicle content‐mixing assay. a) Schematic diagram of the ensemble content‐mixing assay between spike‐cell and ACE2‐vesicle. The spike‐cell and ACE2‐vesicle were mixed with 10 U 3C or 5 U Thr protease, and the fluorescence of dequenching signal of SRB was detected. b) Schematic illustration of the spike and its variants in which functional domains and cleavage sites are highlighted. In the mutants, the furin cleavage site (PRRAR) was mutated into the 3C cleavage site (LEVLFQGP), while the TMPRSS2 cleavage site (KPSKR) was mutated into the Thr cleavage site (LVPRGS). Meanwhile, the C‐terminal cytoplasmic domain of spike^1273^ was deleted to obtain spike variants spike^1268^ and spike^1234^. TM, transmembrane domain; CT, cytoplasmic tail. c) Western blot analysis of the cleavage state of spike^1273^ on the surface of HEK293T cells, which was cleaved by the specified protease. Spike fragments were detected using anti‐RBD antibody targeting the N‐terminal spike and anti‐strep antibody targeting the C‐terminal spike. d) The ensemble content mixing of the spike^1273^‐cell with ACE2‐vesicle. The fluorescence change of SRB for content mixing was normalized with respect to the fluorescence intensity obtained by adding 0.1% Triton X‐100. Traces represent the mean ± SEM from multiple repeats of independent experiments. e) Box plots and data points show maximum fusion probability corresponding to panel d. f) FCM shows the expression level of spike^1273^, spike^1268^ and spike^1234^ on the HEK293T cell surface. The monoclonal antibody 1A9 was used to target S2, and the expression signal from allophycocyanin (APC) that labeled the secondary antibody was detected. g) Box plots and data points show the percentage of spike‐positive cells corresponding to panel f. h) Western blot analysis of the expression of spike^1273^, spike^1268^ and spike^1234^ determined using anti‐RBD primary antibody. i) The ensemble content mixing of the spike‐cell with ACE2‐vesicle in the presence of Thr protease. Traces represent mean ± SEM from multiple repeats of independent experiments. j) Box plots and data points show the maximum fusion probability corresponding to panel i. In panels e, g and j, the repeats (N) of independent experiments were shown above the x‐axis, and statistical analysis was performed using two‐way ANOVA followed by Tukey's multiple comparisons test.

To investigate the mechanism of spike‐mediated viral entry in vitro, we developed an ensemble cell–vesicle content‐mixing assay. In this assay, HEK293T cells were transfected with the plasmid encoding spike (i.e., spike‐cell) to mimic the viral membrane, while ACE2 was reconstituted onto a group of liposomes containing sulforhodamine B (SRB) via a 6×histidine_nickel‐nitrilotriacetic acid (Ni‐NTA) interaction (i.e., ACE2‐vesicle) to mimic the host cell membrane (Figure S1, Supporting Information). Membrane fusion occurred when the spike‐cell and ACE2‐vesicle were mixed (**Figure** **1**a). Content mixing between the spike‐cell and ACE2‐vesicle was monitored based on the increase in the dequenching signal of SRB after fusion.

We firstly expressed the wild‐type spike (spike^WT^) in HEK293T cells. As detected by western blot assay, spike^WT^ was digested into S1 and S2 subunits by an endogenous protease during expression (Figure S2a, Supporting Information). When we mixed the spike^WT^‐cell with the ACE2‐vesicle, mild content mixing was observed in the absence of furin or TMPRSS2 protease (Figure S2b,c, Supporting Information). The inclusion of furin or TMPRSS2 protease resulted in a slight increase in fusion probability; however, there was no significant difference in fusion probability between furin and TMPRSS2 (Figure S2b,c, Supporting Information), which might be due to the pre‐cleaved spike^WT^ masking out the effect of cleavage at the S2’ site by TMPRSS2.

To fully reconstitute spike‐mediated viral entry, it is necessary to obtain an uncleaved spike. The crystal structure of spike revealed that the S1/S2 and S2’ cleavage sites are flexible loops and exposed to the outside (Figure S3, Supporting Information). Theoretically, those endogenous cleavage sites can be replaced without affecting its overall structure and function. Therefore, to prevent auto‐cleavage during expression, we substituted the endogenous S1/S2 and S2’ cleavage sites with 3C and Thr cleavage sites (spike^1273^), respectively (Figure 1b). Western blot analysis was performed to detect the cleavage state of spike^1273^ using an anti‐RBD antibody targeting the S1 subunit and an anti‐strep antibody targeting the C‐terminal strep tag of spike^1273^. As shown in Figure 1c, 3C proteases specifically cleaved spike^1273^ into S1 and S2 subunits, whereas Thr protease cleaved spike^1273^ into S1’ and S2’ subunits.

As a control, when the uninfected‐cell or spike^1273^‐cell was mixed with the ACE2‐vesicle, mild content mixing was observed in the absence of Thr or 3C protease (Figure 1d,e), which might have resulted from the nonspecific uptake of blank‐vesicle by HEK293T cells after 30 min of incubation (Figure S4, Supporting Information). When the S2’ site was cleaved by Thr protease, the fusion probability of content mixing between the spike^1273^‐cell and ACE2‐vesicle was significantly increased, whereas the cleavage of S1/S2 by the 3C protease did not remarkably promote the fusion probability (Figure 1d,e). This suggests that the exposure of FP via cleavage at the S2’ site by Thr is important for spike‐mediated membrane fusion. Moreover, by adding both 3C and Thr proteases, there was no further increase in the fusion probability compared to that of the addition of Thr protease only (Figure 1d,e).

To further improve the in vitro content‐mixing assay, we designed two spike variants (spike^1268^ and spike^1234^) by deleting the C‐terminus of spike^1273^ to increase the localization of spike on the cell surface^[^ ^32^ ^]^ (Figure 1b). In the flow cytometry measurement (FCM) experiments, the expression of both spike^1268^ and spike^1234^ significantly increased on the cell surface (Figure 1f,g), this result was subsequently verified by western blotting (Figure 1h). Consistently, in the presence of Thr protease, both spike^1268^ and spike^1234^ increased the fusion probability of content mixing compared to that of spike^1273^(Figure 1i,j). Similar results were obtained when comparing spike^WT^ and spike^WT1234^ (Figure S2d–h, Supporting Information). Considering that the cluster of cysteine residues at the C‐terminal end of spike may play a role in spike‐mediated membrane fusion,^[^ ^28^, ^33^ ^]^ we mainly presented the results of spike^1268^ in the following experiments, and Thr protease was included in all subsequent experiments unless otherwise specified.

Under physiological conditions, the spike‐localized viral particle is much smaller than the host cell where ACE2 is anchored. In the cell–vesicle content‐mixing assay, we reversed the location of spike and ACE2 by expressing ACE2 on the surface of HEK293T cell and reconstituting purified spike^1268^ onto a group of liposomes (Figure S5a, Supporting Information). We obtained similar enhancement effect on the fusion probability by cleavage at the S1/S2 and S2’ sites (Figure S5b,c, Supporting Information), excluding the possibility that the membrane morphology and local membrane curvature may have an effect on spike‐mediated membrane fusion. However, because of the low yield of spike during purification, we performed the ensemble content‐mixing assay by using the spike‐cell and ACE2‐vesicle in the following experiments. A recent study suggests that low pH is critical for spike‐mediated viral entry into the endocytic pathway.^[^ ^34^ ^]^ However, we did not observe a profound enhancement effect of pH on spike^1268^‐mediated membrane fusion, and low pH even hindered the fusion probability (Figure S6, Supporting Information). This suggests that the direct fusion pathway of viral entry might be pH‐independent, although we cannot rule out the possibility that overexpression of spike^1268^ could mask the effect of pH on membrane fusion.

### 2.2 Binding Affinity of RBD_ACE2 Positively Correlates with the Efficiency of Spike‐Mediated Membrane Fusion

**Figure 2:**
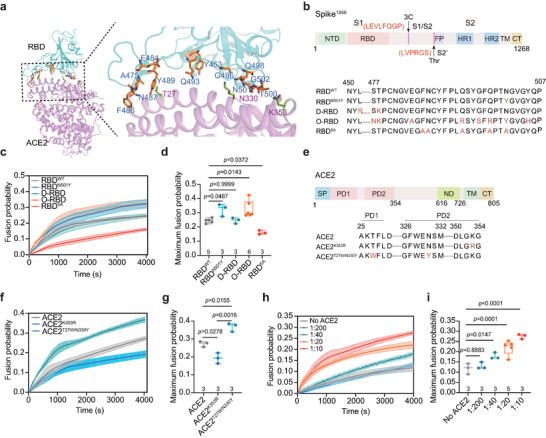
RBD binding to ACE2 is critical for spike‐mediated membrane fusion. a) The interface of the RBD and ACE2 protein (PDB:6LZG) shown in the ribbon diagram, in which RBD and ACE2 are colored in cyan and purple respectively. The key residues that involved in the interface of the RBD and ACE2 were labeled. b) Schematic diagram of the spike^1268^ and mutations on the RBD domain are shown. c) The ensemble content mixing of the spike^1268^ or its mutants‐expressing HEK293T cells with ACE2‐vesicle in the presence of 5 U Thr protease. Traces represent mean ± SEM from multiple repeats of independent experiments. d) Box plots and data points show the maximum fusion probability corresponding to panel c. e) Schematic diagram of ACE2. The mutations on ACE2 are shown as red. SP, signal peptide; PD, peptidase domain; ND, neck domain; TM, transmembrane domain; CT, cytoplasmic tail. f) The ensemble content mixing of the spike^1268^‐cell with vesicles reconstituted with ACE2 or its mutants in the presence of 5 U Thr protease. Traces represent mean ± SEM from multiple repeats of independent experiments. g) Box plots and data points show the maximum fusion probability corresponding to panel f. h) The ensemble content mixing of the spike^1268^‐cell with ACE2‐vesicle at different molar ratios of ACE2: lipids in the presence of 5 U Thr protease. Traces represent mean ± SEM from multiple repeats of independent experiments. i) Box plots and data points show the maximum fusion probability corresponding to panel h. In panels d, g, and i, the repeats (N) of independent experiments were shown above the x‐axis, and statistical analysis was performed using two‐way ANOVA followed by Tukey's multiple comparisons test.

As the binding of the RBD to ACE2 is critical for spike initiation in viral infections, we investigated whether this interaction between the RBD and ACE2 has functional consequences in spike‐mediated membrane fusion. We first investigated the binding affinity between ACE2 and different RBD mutants derived from the spike variants. Based on the structure of the RBD_ACE2 complex (**Figure** **2**a), we designed a series of RBD mutants: RBD^N501Y^ (originating from the alpha variant),^[^ ^22^ ^]^ D‐RBD (originating from the delta variant),^[^ ^35^ ^]^ O‐RBD (originating from the omicron variant),^[^ ^36^ ^]^ and RBD^5A^, which had five mutated residues that are involved in the interface of the RBD and ACE2^[^ ^15^ ^]^ (Figure 2b). The binding affinity of ACE2 to RBD or its variants was determined using surface plasmon resonance (SPR). Compared to that of RBD^WT^ (7.21 × 10^−8^ m), the binding affinity of RBD^N501Y^ (2.82 × 10^−8^ m), D‐RBD (2.96 × 10^−8^ m), and O‐RBD (1.91 × 10^−8^ m) to ACE2 were enhanced by ≈2‐4 fold (Figure S7, Supporting Information). In contrast, the binding affinity of RBD^5A^ to ACE2 (1.85 × 10^−7^ m) was reduced by ≈3 fold (Figure S7, Supporting Information). Next, using FCM and western blot analysis, we investigated the effect of different RBD variants in the cell–vesicle content‐mixing assay by adjusting the expression of the spike^1268^ and its variants to a similar level (Figure S8, Supporting Information). Consistently, in the ensemble cell–vesicle content‐mixing assay in the presence of Thr protease, RBD^N501Y^ and O‐RBD slightly promoted the fusion probability compared to that of RBD^WT^, whereas the RBD^5A^ variant significantly reduced the fusion probability (Figure 2c,d). Notably, we did not observe a significant effect on fusion probability using D‐RBD (Figure 2c,d). Meanwhile, we also generated two mutants of ACE2 (K353R and T27W/N330Y), which have been reported to affect the binding affinity to RBD^[^ ^23^ ^]^ (Figure 2e), and verified that the binding affinity of ACE2^K353R^ to RBD^WT^ (1.84 × 10^−7^ m) was reduced by ≈2 fold compared to that of ACE2 (7.57 × 10^−8^ m), while the binding affinity of ACE2^T27W/N330Y^ was enhanced by ≈4 fold (1.93 × 10^−8^ m) (Figure S9, Supporting Information). By reconstituting equal amounts of ACE2 and its variants into liposomes (Figure S8d, Supporting Information), we tested the effect of these variants in the cell–vesicle content‐mixing assay in the presence of Thr protease. The results showed that ACE2^T27W/N330Y^, which enhances the binding affinity to RBD also promoted fusion probability, whereas ACE2^K353R^, which reduces the binding affinity to RBD, inhibited fusion probability (Figure 2f,g). Moreover, by changing the ratio of ACE2 to lipid on liposomes from 1:200 to 1:10, we found that the fusion probability increased as the concentration of ACE2 increased, whereas vesicles without ACE2 showed negligible fusion probability (Figure 2h,i), indicating that spike‐mediated membrane fusion is ACE2‐dependent. We also repeated all these experiments using a shorter version of spike^1234^ and confirmed the results (Figure S10, Supporting Information). Taken together, we revealed a positive correlation between the binding affinity of the RBD to ACE2 and the membrane fusion efficiency mediated by spike, indicating that the interaction between the RBD and ACE2 plays an important role in spike‐mediated viral invasion.

### 2.3 Spike Drives Membrane Fusion in the Absence of ACE2 In Vitro

**Figure 3:**
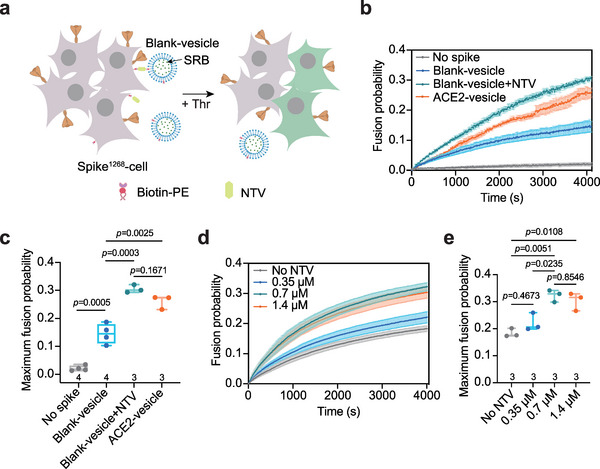
Spike drives membrane fusion by crosslinking two membranes in the cell–vesicle content‐mixing assay. a) Schematic diagram showing the in vitro cell–vesicle content‐mixing assay between spike^1268^‐cell and blank‐vesicle by crosslinking membranes via the NTV_biotin interaction. b) The ensemble cell–vesicle content mixing of the spike^1268^‐cell with ACE2‐vesicle or blank‐vesicle. Traces represent mean ± SEM from multiple repeats of independent experiments in the presence of 5 U Thr protease. c) Box plots and data points show the maximum fusion probability corresponding to panel b. d) The ensemble cell–vesicle content‐mixing assay of the spike^1268^‐cell with blank‐vesicle at specified concentration of NTV in the presence of 5 U Thr protease. Traces represent mean ± SEM from multiple repeats of independent experiments e) Box plots and data points show the maximum fusion probability corresponding to panel d. In Panels c and e, the repeats (N) of independent experiments were shown above the x‐axis, and statistical analysis was performed using two‐way ANOVA followed by Tukey's multiple comparisons test.

Although the interaction of the RBD with ACE2 is essential for spike‐mediated viral entry, alternative membrane proteins, such as AXL and CD147, have also been identified as candidate receptors for spike.^[^ ^29^, ^31^ ^]^ To further investigate whether RBD binding to ACE2 is dispensable for spike‐mediated membrane fusion in the reconstituted fusion system, we used neutravidin (NTV), a deglycosylated native avidin composed of four identical biotin‐binding subunits, to crosslink the biotin‐containing cell and vesicle membranes in the ensemble cell–vesicle content‐mixing assay (**Figure** **3**a). In all experiments, 5 U Thr protease was added to induce cleavage at the S2’ site. When ACE2 was excluded, we observed only mild content mixing between the spike^1268^‐cell and blank‐vesicle. Notably, the fusion probability was considerably promoted when 0.7 µm NTV was included, which is comparable to the fusion probability mediated by the RBD_ACE2 interaction (Figure 3b,c). However, when spike^1268^ was left out, little content mixing was observed in the presence of 0.7 µm NTV (Figure 3b,c), suggesting the membrane fusion is spike‐dependent. Moreover, the fusion probability increased with the NTV concentration, reaching a maximum at a concentration of 0.7 µm NTV (Figure 3d,e). We also repeated these experiments with a shorter version of spike^1234^ and confirmed the results (Figure S11, Supporting Information). This suggests that the RBD_ACE2 interaction may function as a crosslinker to bridge the two membranes.

**Figure 4:**
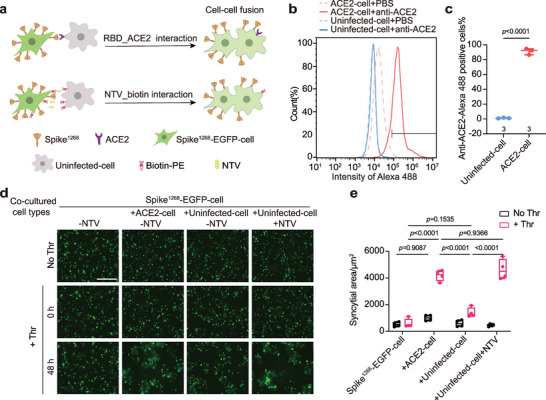
Spike induces syncytia formation by crosslinking two cell membranes. a) Schematic diagram of the cell–cell fusion assay in the presence of ACE2 (upper), or NTV (bottom). b) FCM shows the expression level of ACE2 on the ACE2‐cell and uninfected‐cell. The rabbit polyclonal antibody was used to target ACE2, and the signal of Alexa Fluor 488‐labeled the secondary antibody was detected. c) Box plots and data points show the percentage of ACE2‐positive cells corresponding to panel b. d) Syncytia formation induced by spike^1268^ mediated cell–cell fusion via the NTV_biotin interaction or the RBD_ACE2 interaction. After spike^1268^‐EGFP‐cell, ACE2‐cell, and uninfected‐cell were treated with biotin‐PE, spike^1268^‐EGFP‐cell and ACE2‐cell (or uninfected‐cell) were co‐cultured at a 2:1 ratio with or without 5 U Thr protease. Scale bar, 200 µm. e) Box plots and data points show the area of syncytia in cell–cell fusion corresponding to panel d. The area of syncytia was calculated based on the fused cells with weak fluorescence intensity of EGFP and were at least twice as large as the unfused cells. At least five fields were randomly selected in each well. Statistical analysis was performed using two‐way ANOVA followed by Tukey's multiple comparisons test.

Next, using a cell–cell fusion assay, we examine whether NTV‐crosslinked membranes could also induce spike‐mediated membrane fusion. In this fusion assay, one group of HEK293T cells was co‐transfected with the plasmids encoding EGFP and spike^1268^ (spike^1268^‐EGFP‐cell), whereas the other group of HEK293T cells was transfected with the plasmid encoding human ACE2 (ACE2‐cell), or left untreated (uninfected‐cell) (**Figure** **4**a). When the two groups of cells were cocultured, the syncytia were visualized based on the enlarged area of the EGFP fluorescent reporter if fusion occurred (Figure 4a; Figure S12, Supporting Information). The efficiency of cell–cell fusion was quantified by summarizing the area of the syncytia in each image. We first verified the expression of ACE2 on the surface of the ACE2‐cell, and almost no ACE2 was observed in uninfected‐cell in the FCM experiments (Figure 4b,c). Again, in all experiments, 5 U Thr protease was included to induce cleavage at the S2’ site unless specified, ACE2‐cell formed large multinucleate syncytia when co‐cultured with the spike^1268^‐EGFP‐cell, while uninfected‐cell did not (Figure 4d,e; Figure S12, Supporting Information).

To crosslink cell membranes, both the spike^1268^‐EGFP‐cell and uninfected‐cell groups were cultured with 1 µm biotin‐PE. As a control, co‐culture of the biotin treated spike^1268^‐EGFP‐cell and uninfected‐cell did not form large syncytia in the absence of NTV (Figure 4d,e). When 1 µm NTV was included, large multinucleate syncytia were formed between the biotin treated spike^1268^‐EGFP‐cell and uninfected‐cell, and the area of syncytia induced by NTV was comparable to that mediated by ACE2 (Figure 4d,e). We repeated these experiments with a shorter version of spike^1234^ and confirmed these results (Figure S13, Supporting Information). In summary, our results suggest the RBD_ACE2 interaction may function as a crosslinker to pull the viral and cell membranes closer, and can be substituted by other membrane cross‐linkers.

### 2.4 A Suitable Distance between Two Membranes is Critical for Spike‐Mediated Membrane Fusion

**Figure 5:**
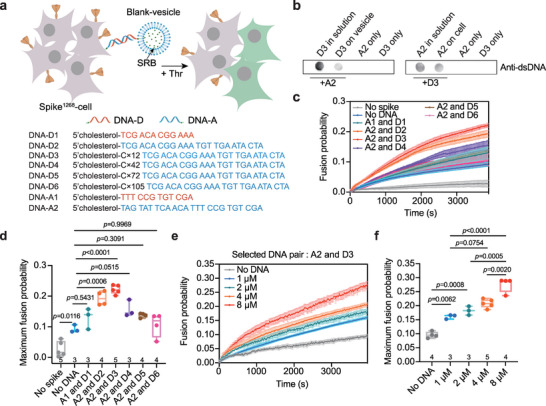
Spike promotes membrane fusion at an optimal distance between two membranes in the cell–vesicle content‐mixing assay. a) Schematic diagram showing the in vitro cell–vesicle content‐mixing assay between the spike^1268^‐cell and blank‐vesicle by crosslinking membranes via lipid‐anchored complementary DNA strands. Cholesterol‐linked DNA‐A was reconstituted onto spike^1268^‐cell, while cholesterol‐linked DNA‐D was reconstituted onto vesicles. The complementary DNA pairs are colored in red and blue respectively. b) Dot‐blot assay shows the efficiency of the reconstitution of DNA strands (A2 and D3) onto the vesicle and cell membrane. When DNA strands on the membrane binds to its complementary pair of DNA strands, they were detected by DNA primary antibody that targeting on DNA double helix. “in solution” indicates DNA strands that were not reconstituted. “on vesicle” and “on cell” indicate DNA strands that were reconstituted onto the vesicle membrane and cell membrane, respectively. c) The ensemble cell–vesicle content‐mixing assay of DNA‐A contained spike^1268^‐cell with DNA‐D contained blank‐vesicle by using different lengths of DNA strands in the presence of 5 U Thr protease. Traces represent mean ± SEM from multiple repeats of independent experiments. d) Box plots and data points show the maximum fusion probability corresponding to panel c. e) The ensemble cell–vesicle content‐mixing assay of the spike^1268^‐cell with blank‐vesicle at specified concentration of DNA strands (A2 and D3). Traces represent mean ± SEM from multiple repeats of independent experiments. f) Box plots and data points show the maximum fusion probability corresponding to panel e. In Panels d and f, the repeats (N) of independent experiments were shown above the x‐axis, and statistical analysis was performed using two‐way ANOVA followed by Tukey's multiple comparisons test.

To investigate whether there is an optimal distance to initiate spike‐mediated viral invasion, we designed a series of complementary DNA strands of different lengths to crosslink the spike^1268^‐cell and vesicle membranes. The acceptor DNA strands (DNA‐A) with a cholesterol tail were reconstituted on the cell membrane, whereas the donor DNA strands (DNA‐D), which contained a complementary DNA sequence corresponding to DNA‐A and a polycytosine deoxynucleotide linker, were reconstituted on the vesicle membrane via a cholesterol anchor (**Figure** **5**a,b). We then examined the effect of DNA strands length in a cell–vesicle content‐mixing assay in the presence of 5 U Thr protease. In the control, negligible fusion was observed upon mixing the spike^1268^‐cell and blank‐vesicle in the absence of complementary DNA strands (Figure 5c,d). Notably, when the short DNA strands of DNA‐A1 (12 bp) and DNA‐D1 (12 bp) were incorporated into the spike^1268^‐cell and blank‐vesicle, respectively, the fusion probability did not increase significantly (Figure 5c,d). However, when the membranes were crosslinked with the DNA strands DNA‐A2 (24 bp) and DNA‐D2 (24 bp), the fusion probability was significantly enhanced (Figure 5c,d). The length of DNA‐D was gradually increased from 12 bp (DNA‐D3) to 105 bp (DNA‐D6) by adjusting the poly‐cytosine deoxynucleotide linker. The results revealed that DNA‐D3 (36 bp) significantly promoted spike‐mediated membrane fusion, whereas the effect of the longer DNA‐D strands was negligible (Figure 5c,d). As a control, when spike^1268^ was left out, the complementary DNA strands of DNA‐A2 and DNA‐D3 did not induce membrane fusion (Figure 5c,d). By reconstituting different concentrations of DNA‐A2 onto the cell membrane and DNA‐D3 onto the vesicle membrane, we found that the fusion probability increased as a function of the DNA concentration on the membrane (Figure 5e,f). Again, we selected two pairs of DNA strands and repeated these experiments with a short version of spike^1234^ (Figure S14, Supporting Information). Taken together, the ensemble content‐mixing assay revealed that an optimal distance between the virus membrane and the cell membrane might be required for spike to initiate membrane fusion.

**Figure 6:**
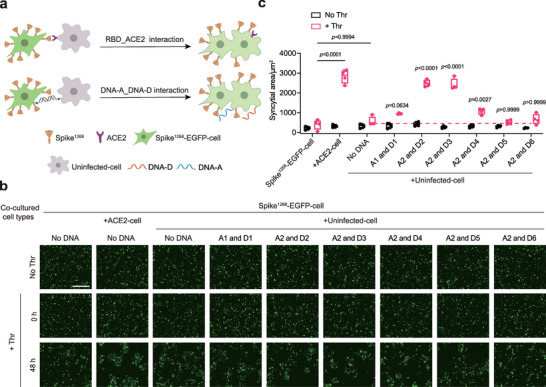
Spike induces syncytia formation at an optimal distance of cell membranes. a) Schematic diagram of the cell–cell fusion assay in the presence of ACE2 (upper), or lipid‐anchored complementary DNA strands (bottom). Cholesterol‐linked DNA‐A was reconstituted onto spike^1268^‐EGFP‐cell, while cholesterol‐linked DNA‐D was reconstituted onto uninfected‐cell. b) Syncytia formation was induced by spike^1268^ mediated cell–cell fusion. Spike^1268^‐EGFP‐cell containing DNA‐A was co‐cultured with uninfected‐cell containing specified DNA‐D at a 2:1 ratio. Scale bar, 200 µm. c) Quantitative analysis of the area of syncytia in cell–cell fusion shown in b, statistical analysis was performed using two‐way ANOVA among the experiments in the presence of Thr, and the statistical analysis within dashed line was compared to the experiment without DNA strands followed by Tukey's multiple comparisons test.

Next, we examined whether the distance between the crosslinked membranes was important for spike mediated cell–cell fusion. As shown in Figure 4, we prepared three groups of HEK293T cells: spike^1268^‐EGFP‐cell, ACE2‐cell and uninfected‐cell. To crosslink the cell membrane, the spike^1268^‐EGFP‐cell was cultured with cholesterol‐linked DNA‐A, whereas uninfected‐cell were cultured with cholesterol‐linked DNA‐D (**Figure** **6**a). In all experiments, 5 U Thr protease was included to induce cleavage at the S2’ site unless specified. In the control, large multinucleated syncytia were not formed, when the spike^1268^‐EGFP‐cell and uninfected‐cell groups were co‐cultured (Figure 6b,c). Consistently, when the two groups of cells were crosslinked with a short DNA pair (DNA‐A1 and DNA‐D1), few multinucleated syncytia were observed (Figure 6b,c). The formation of multinucleated syncytia was remarkably enhanced only when the cell membranes were crosslinked by a pair of DNA‐A2 and DNA‐D2 or a pair of DNA‐A2 and DNA‐D3 but not by other longer DNA strands (Figure 6b,c; Figure S12, Supporting Information). Moreover, the area of the multinucleated syncytia induced by coculturing spike^1268^‐EGFP‐cell and ACE2‐cell was comparable to that by coculturing the spike^1268^‐EGFP‐cell and uninfected‐cell groups crosslinked with a suitable pair of DNA strands (Figure 6b,c). In the absence of the Thr protease, no multinucleated syncytia were formed under any of the conditions, indicating that the formation of syncytia depends on the cleavage of the spike at the S2’ site (Figure 6b,c). In summary, these results suggest that RBD interacting with ACE2 may provide a suitable distance between the viral and the host cell membranes, which might be required for spike‐mediated viral invasion.

### 2.5 Inhibition of Spike‐Mediated Membrane Fusion by Manipulating the Distance Between Two Membranes

**Figure 7:**
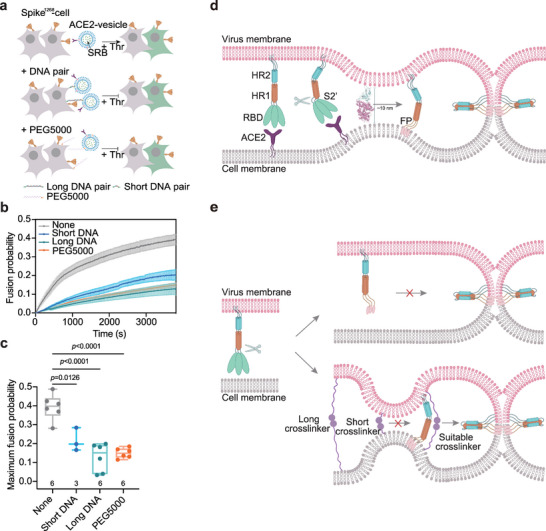
Spike‐mediated membrane fusion can be inhibited by manipulating the distance between two membranes. a) Schematic diagram showing the inhibition of spike mediated membrane fusion between spike^1268^‐cell and ACE2‐vesicle by altering the distance between two membranes. A pair of cholesterol linked complementary DNA (long DNA pair [129 bp]; short DNA pair [6 bp]) or PE‐linked PEG5000 was reconstituted onto spike^1268^‐cell and ACE2‐vesicle. b) The ensemble cell–vesicle content‐mixing assay of the spike^1268^‐cell with ACE2‐vesicle in the presence of specified membrane crosslinker in the presence of 5 U Thr protease. c) Box plots and data points show the maximum fusion probability corresponding to panel b. d) Spike mediated viral invasion initiated by the distance regulator of the RBD_ACE2 interaction. Viral invasion is induced by the formation of six‐helix bundle of HR1 and HR2, following the action of cleavage at the S2’ site. e) Spike mediated viral invasion initiated by other distance regulators. In the absence of ACE2, spike alone does not drive viral invasion. However, when membrane crosslinker bridges two membranes at a suitable distance, spike is capable of driving viral invasion in the absence of ACE2. Statistical analysis was performed using two‐way ANOVA followed by Tukey's multiple comparisons test.

Finally, we investigated whether spike‐mediated membrane fusion could be prevented by altering the distance between the two membranes. To test this hypothesis, we increased the distance between the two membranes by including phosphoethanolamine (PE)‐linked methoxy (polyethylene glycol)−5000 (PEG5000) or a pair of complementary DNA strands (129 bp) on the spike^1268^‐cell and ACE2‐vesicle (**Figure** **7**a). We shortened the distance between the two membranes using a pair of short complementary DNA strands (6 bp) (Figure 7a). In the absence of membrane distance manipulation, when the spike^1268^‐cell and ACE2‐vesicle were mixed in the presence of the Thr protease, robust fusion occurred (Figure 7b). Notably, when PEG5000 or a pair of long complementary DNA strands was incorporated into the spike^1268^‐cell and ACE2‐vesicle, the fusion was significantly inhibited by approximately 65% (Figure 7b,c). Moreover, when the two membranes were tightly crosslinked by a short DNA pair, spike^1268^ mediated membrane fusion was markedly inhibited by approximately 48% (Figure 7b,c). Taken together, our results suggest that spike‐mediated membrane fusion could be inhibited by manipulating the membrane distance, which may provide new insights for developing inhibitors of SARS‐CoV‐2 infection.

## 3 Discussion

During the invasion of SARS‐CoV‐2, spike‐mediated membrane fusion relies on the recognition of ACE2 by the RBD.^[^ ^1^, ^10^ ^]^ To investigate the function of the interaction between the RBD and ACE2, we developed an in vitro ensemble cell–vesicle content‐mixing assay to mimic SARS‐CoV‐2 invasion. In this assay, we substituted the furin and TMPRSS2 endogenous cleavage sites with 3C and Thr cleavage sites to prevent auto‐cleavage of spike during expression. The engineered spike^1273^ considerably promoted membrane fusion between spike^1273^‐cell and ACE2‐vesicle by the action of Thr protease on the S2’ site but not by the action of 3C protease on the S1/S2 site, (Figure 1c–e). Although the unique furin‐like (S1/S2) cleavage site has been speculated to be responsible for the high infectivity and transmissibility of SARS‐COV‐2.^[^ ^37^ ^]^ previous studies have shown that mutagenesis of the S2’ cleavage site, but not the S1/S2 cleavage site, is sufficient to prevent spike‐mediated viral infection,^[^ ^17^ ^]^ and the exposure of FP driven by the conformational transitions of HR1 leads to its insertion into the host cell membrane.^[^ ^12^, ^38^ ^]^ In our reconstituted fusion system, the cleavage of S2’ by Thr protease seems to be more important than the cleavage at S1/S2 by 3C protease in spike‐mediated membrane fusion. This supports the notion that the conformational change of spike and the exposure of FP induced by the cleavage at the S2’ site play a critical role in spike‐mediated viral invasion.

We further improved the fusion assay by deleting the C‐terminal cytoplasmic part of spike to increase its expression on the cell surface, thereby further increasing the fusion efficiency (Figure 1f–j). It has been reported that the poly‐cysteine region in the C‐terminal cytoplasmic part of spike may be involved in the interaction with cholesterol in the viral membrane, although its functional consequence is not yet clear.^[^ ^28^, ^33^, ^39^ ^]^ However, we did not observe a significant difference between spike^1268^ and spike^1234^ in fusion efficiency under our experimental conditions, suggesting that the role of the poly‐cysteine region in the C‐terminal tail of spike may not be in the viral membrane fusion but instead is in the packaging of spike into budding virions in cholesterol‐rich lipid microdomains.^[^ ^33^ ^]^

Association between the viral membrane and host cell is initiated by the interaction between the RBD and ACE2. Normally, the rate of membrane association predominantly affects the capacity of fusogenic protein‐mediated membrane fusion.^[^ ^40^, ^41^ ^]^ Next, we examined the effect of the RBD_ACE2 interaction on spike‐mediated membrane fusion. Based on the variants of SARS‐CoV‐2 that have appeared during evolution,^[^ ^22^, ^35^, ^36^ ^]^ we generated the mutations at the interface between the RBD and ACE2 (Figure 2). Mutations in both the RBD and ACE2 resulted in substantial changes in their binding affinity (Figure S7 and S9, Supporting Information), and the change in the binding affinity of the RBD to ACE2 positively correlated with the efficiency of spike variants‐mediated membrane fusion (Figure 2). Thus, our results may explain why the high infectivity of the variants of SARS‐COV‐2 may result from the high binding affinity between the RBD and ACE2.

The discovery of alternative receptors raised the question of whether the binding of RBD to ACE2 is indispensable for spike‐mediated viral invasion.^[^ ^29^, ^31^ ^]^ To address this question, we crosslinked the cell membrane and the vesicle membrane via the NTV_biotin interaction (Figure 3 and 4) or complementary DNA pairs (Figure 5 and 6), and demonstrated that cross‐linking membranes were sufficient for spike to initiate membrane fusion (Figure 3, 4, 5, 6), Notably, although our results suggest that ACE2 could be omitted in spike‐mediated in vitro fusion assays when two membranes are crosslinked, under physiological conditions, ACE2, as the predominant receptor on the host cell surface, is still required for spike‐mediated viral invasion. Finally, using a series of complementary DNA pairs of different lengths, we studied the effect of crosslinker length on spike‐mediated viral invasion. In both the cell–vesicle content‐mixing and the cell–cell fusion assays, the results revealed that the DNA strands of 24–36 bp showed the strongest enhancement in spike‐mediated membrane fusion (Figure 5, 6). Given a length of 0.34 nm per base pair in the DNA double helix, the optimal length of the DNA crosslinker is estimated ≈8–12 nm (Figure 7), which is comparable to the length of the RBD_ACE2 complex (≈10 nm).^[^ ^15^ ^]^ Thus, our results indicate that the function of RBD binding to ACE2 may be to provide a suitable distance for the spike to initiate membrane fusion.

Prevention of SARS‐CoV‐2 infection by interfering with the interaction between the RBD and ACE2 is one of the main therapeutic approaches.^[^ ^23^, ^42^, ^43^, ^44^ ^]^ Although effective strategies targeting the RBD_ACE2 interaction, including therapeutic agents, vaccines, and neutralizing antibodies,^[^ ^45^, ^46^, ^47^, ^48^, ^49^ ^]^ show an exceptional ability to block SARS‐CoV‐2 infection, they may not offer complete protection against a virus that mutates rapidly during evolution.^[^ ^50^ ^]^ Therefore, the development of broad‐spectrum inhibitors appears to be advantageous. Our data showed that by manipulating the distance between the two membranes, spike‐mediated membrane fusion was effectively inhibited without interfering with the RBD_ACE2 interaction (Figure 7a–c). This could be used as an alternative strategy to prevent SARS‐CoV‐2 invasion. Membrane‐binding proteins or polypeptides that bear multiple membrane‐binding sites via oligomerization can be selected as potential membrane crosslinkers to tightly bridge viral particles and host cells at a shorter distance than that regulated by the RBD_ACE2 interaction.^[^ ^51^, ^52^ ^]^ However, without intensive validation and precise control of the membrane distance, it might be unrealistic and insecure to apply such membrane crosslinkers as therapeutic inhibitors of viral infection.

In summary, our results suggest that the interaction of the RBD with ACE2 may function as crosslinker to bridge the viral and cell membrane at a suitable distance for spike to initiate viral invasion (Figure 7d). When ACE2 was excluded, membrane fusion between the virus and host cell was prevented because the released FP could not reach the cell membrane effectively (Figure 7e). However, membrane fusion occurred even in the absence of ACE2, when the viral membranes were crosslinked at a suitable distance (Figure 7e). From a broad perspective, highly transmissible human coronaviruses share a conserved mechanism of viral entry, and manipulation of the membrane distance might be a new strategy for the development of broad‐spectrum antiviral inhibitors.

## 4 Experimental Section

### Plasmid Cloning, Expression, and Purification

The genes encoding spike^WT^ (20‐1273), spike^1273^(20‐1273), spike^1268^(20‐1268), and spike^1234^ (20‐1234) were cloned into the PTT5 vector. The Kozak sequence (GCCACC) and signal peptide (METDTLLLWVLLLWVPGSTG) were added to the N‐terminus of the spike or its variant, and a twin‐strep tag was added to the C‐terminus. For the mutants spike^1273^, spike^1268^, and spike^1234^, the residues within the S1/S2 cleavage site (PRRAR) were replaced with the 3C cleavage site (LEVLFQGP), and the residues within the S2’ cleavage site (KPSKR) were replaced with the Thr cleavage site (LVPRGS). Recombinant bacmids of His‐tagged RBD (residues 320–537), RBD variants (RBD^N501Y^, D‐RBD, O‐RBD, and RBD^5A^), human ACE2 (residues 19–615), or ACE2 variants (ACE2^T27W/N330Y^and ACE2^K353R^) with an N‐terminal GP67 signal peptide were transfected into sf9 insect cells using the Bac‐to‐Bac baculovirus expression system. Briefly, 6 µL Cellfectin (Invitrogen) was diluted in 100 µL SIM SF medium (Sino Biological), and the resulting mixture was then mixed with another 100 µL sf9 SIM SF medium containing 1 µg recombinant bacmid. After 20 min of incubation, 1800 µL of SIM SF medium was added to the mixture and subsequently, transferred into 8 × 10^5^ sf9 cells in 6‐well plates (BIOFIL). After 5 h of incubation, the cell supernatant was replaced with 2 mL of fresh SIM SF medium containing 100 U/mL penicillin (Beyotime) and 100 µg mL^−1^ streptomycin (Beyotime). After another 72 h of incubation, the recombinant baculoviruses in the supernatant were harvested by centrifugation at 1500 rpm for 5 min. Sf9 cells (800 mL) in the logarithmic growth phase (2.0 × 10^6^ cells mL^−1^) were infected with the recombinant baculovirus for 72 h; then, the infected sf9 cells were harvested by centrifugation at 8000 rpm for 30 min.

Next, affinity chromatography using Ni‐NTA (Smart‐Lifesciences) was used to purify ACE2, RBD, and their mutants. The details of protein purification have been described in a previous study.^[^ ^23^ ^]^ ACE2 and its mutants were loaded on Source 15Q column (GE Healthcare) for ion exchange, followed by size exclusion using the Superdex 200 Increase 10/300 GL column (GE Healthcare). The RBD and its mutants were loaded onto a Source SP HP column (GE Healthcare) for ion exchange, followed by size exclusion using the Superdex 200 Increase 10/300 GL column. Protein purity was determined using SDS‐PAGE and visualized by Coomassie blue staining.

For the expression and purification of spike^1268^, Expi293F cells were cultured with 293‐II serum‐free medium (SinoBiological) containing 100 U/mL penicillin (Beyotime) and 100 µg mL^−1^ streptomycin (Beyotime). 800 mL Expi293F cells at a density of 2–3 × 10^6^ mL^−1^ were transiently transfected with the 800 µg plasmid of spike^1268^ containing 2.6 mg polyethylenimine (Polyscienes). The transfected cells were harvested 48–60 h after transfection at a density of 4–5 × 10^6^ mL^−1^ by centrifugation for 30 min at 4000 rpm at 4 °C. Then the cell pellet was washed two rounds and resuspended in PBS (135 mm NaCl, 4.7 mm KCl, 10 mm Na_2_HPO_4_, 2 mm NaH_2_PO_4_, pH 7.4) (BOSTER), supplemented with 10 mm PMSF (Sangon Biotech) and EDTA‐free complete protease inhibitor cocktail (Beyotime). Next, after the lysis of cells by sonication, the cell debris was removed by centrifugation for 30 min at 10000 rpm, and the supernatant was centrifuged at 40000 rpm for 1 h to pellet the membrane. Subsequently, the pellet was resuspended with PBS, supplemented with 1 mM TECP, 2% DDM (LABLEAD) and solubilized at 4  °C with stirring for 30 min. The solubilized membrane was centrifuged at 40000 rpm for 40 min, then the supernatant was mixed with 2 ml strep‐tactin (Smart Lifescience) beads and incubated for 4 °C for 30 min, the beads were washed with PBS, supplemented with 0.8% OG (LABLEAD) and then eluted with PBS containing 0.8% OG and 50 mM desthiobiotin (Sangon Biotech). Finally, protein‐containing fractions were combined, the protein concentration was measured by absorption at 280 nm and aliquots were flash‐frozen in liquid nitrogen and stored at −80 °C.

### Reconstitution of ACE2‐Vesicle

5 µmole lipids mixture was dissolved into chloroform solution in a glass tube, and dried the lipids under a gentle stream of argon inside a chemical fume hood. The lipids mixture consists of 14% DOPS (1,2‐dioleoyl‐*sn*‐glycero‐3‐phospho‐L‐serine), 41% POPC (1‐palmitoyl‐2‐oleoyl‐*sn*‐glycero‐3‐phosphocholine), 18% Cholesterol, 18% DOPE (1,2‐dioleoyl‐*sn*‐glycero‐3‐phosphoethanolamine), 9% DGS‐Ni‐NTA (1,2‐dioleoyl‐*sn*‐glycero‐3‐[(N‐(5‐amino‐1‐carboxypentyl) iminodiacetic acid) succinyl] (nickel salt)). 1% Biotin‐PE (1‐oleoyl‐2‐(12‐biotinyl(aminododecanoyl))‐*sn*‐glycero‐3‐phosphoethanolamine), 1% PE linked PEG5000 (1,2‐dipalmitoyl‐*sn*‐glycero‐3‐phosphoethanolamine‐N‐[methoxy(polyethylene glycol)−5000]), or 1% cholesterol linked DNA strands was included by replacing equal amount of POPC when specified. All lipids are purchased from Avanti Polar Lipids. For the preparation of ACE2‐vesicle, the lipid mixture was resuspended in vesicle buffer containing 20 mm SRB (Invitrogen) to make the total lipid concentration 10 m. After ten freeze–thaw cycles, unilamellar proteoliposomes were extruded through polycarbonate filters with 50 nm pore size (Whatman) at least 31 times. Then 40 µm his‐tagged ACE2 was mixed with 50 µL blank‐vesicle and then incubated at 4 °C overnight. The ACE2‐vesicle was subsequently purified through CL‐4B column. Collected fraction of ACE2‐vesicle was confirmed by 10% SDS‐PAGE. For the preparation of spike^1268^‐vesicle, 1 µm spike^1268^ was mixed with 50 µL blank‐vesicle which was supplemented with 0.8% OG, and the concentration of OG was kept at 0.8% during the reconstitution. After 4 °C incubation for 20 min, the protein/lipid mixtures were diluted two times to make the concentration of OG below the critical micelle concentration (≈0.6%). The mixtures were then dialyzed overnight against vesicle buffer at 4 °C. All cholesterol linked DNA strands were synthesized from Sangon Biotech (Table S1).

### Negative‐Staining Transmission Electron Microscopy Imaging

ACE2‐vesicle was applied to a continuous carbon‐coated EM grid, which was glow‐discharged (PELCO easiGlow) for 45 s. After 1 min incubation, the grid was blotted dry with filter paper, and stained with 1% uranyl acetate (Electron Microscopy Sciences) and air‐dried. The negatively stained specimens were examined on a transmission electron microscope (JEOL1400) and operated at an acceleration voltage of 120 kV.

### Cell Culture

HEK293T cells were cultured in Dulbecco's Modified Eagle Medium (DMEM) (GE Healthcare) supplemented with 10% fetal bovine serum (FBS), 100 U mL^−1^ penicillin, and 100 µg mL^−1^ streptomycin at 37 °C under 5% CO_2_ in an incubator. Expi293F cells were cultured in 293‐II serum‐free medium supplemented with 100 U mL^−1^ penicillin and 100 µg mL^−1^ streptomycin at 37 °C under 5% CO_2_ in an incubator. Sf9 cells were cultured in SIM SF medium supplemented with 100 U mL^−1^ penicillin and 100 µg mL^−1^ streptomycin in a non‐humidified shaker at 27 °C. All cells were purchased from the American Type Culture Collection (ATCC).

### Cell Transient Transfection

Before transfection, the HEK293T cells were cultured for 24 h to reach the logarithmic growth phase. For transient transfection, 24 µL Lipo8000 transfection reagent (Beyotime) was added into 750 µL DMEM containing 15 µg of a plasmid encoding a specific protein, followed by 48 h of culture. Cells were collected after three rounds of washing with PBS buffer for further experiments.

### Western Blot

Cells were lysed for 5 min by sonication at the power of 300 w on ice in RIPA buffer (50 mm Tris, 150 mm NaCl, 1% Triton X‐100, 1% sodium deoxycholate, 0.1% SDS, pH 7.4) (Sigma), supplemented with 10 mm PMSF (Sangon Biotech) and EDTA‐free complete protease inhibitor cocktail (Beyotime). The supernatant was collected by centrifugation at 15 000 rpm for 20 min at 4 °C and total protein concentrations were determined using the BCA protein assay kit (Thermo Fisher Scientific). Subsequently, equal amounts (20 µg) of cell lysates were loaded onto a 10% SDS‐PAGE under reducing conditions and were transferred onto a 0.22 µm polyvinylidene difluoride (PVDF) membrane (Millipore). The membrane was blocked with 5% (w/v) skim milk (Sangon Biotech) in TBST (20 mm Tris, 137 mm NaCl, 0.1% Triton X‐100, pH 7.6) (BOSTER) for 1 h at room temperature. Next, the membrane was incubated with rabbit polyclonal anti‐RBD antibody (1:2500 dilution) (SinoBiological), 400 ng mL^−1^ mouse monoclonal anti‐b‐actin antibody (ZSGB‐BIO) or rabbit polyclonal anti‐S2 antibody (1:2500 dilution) (SinoBiological) in TBST at 4 °C overnight, followed by three rounds of washing with TBST at room temperature for 15 min. It was further incubated with 80 ng mL^−1^ HRP‐conjugated goat anti‐rabbit/mouse IgG(H+L) (ZSGB‐BIO) for 2 h at 37 °C. Finally, after three rounds of washing with TBST, the PVDF membranes were visualized using a chemiluminescent HRP substrate (Millipore). Immunoblot images were digitally captured using the Tanon‐5200 chemiluminescent imaging system. To test the cleavage state of spike^1273^, 3C (Beyotime) and Thr (Sigma) protease at specified concentration were added to 50 µL cell culture for 2 h at 37 °C. Equal amounts of cell culture (8 µL) were loaded onto 10% SDS‐PAGE gels and transferred onto PVDF membranes. The cleavage state of spike was visualized using both the anti‐RBD antibody and the anti‐Strep antibody using the same aforementioned steps.

### Ensemble Cell–Vesicle Content‐Mixing Assays

For the ensemble cell–vesicle content‐mixing assays, the ACE2‐vesicle or blank‐vesicle containing 20 mm self‐quenched SRB was used as a content indicator. Content mixing was measured in the vesicle buffer based on an increase in fluorescence emission at 585 nm upon excitation with a 532 nm laser light. The dequenching signal resulted from dilution of the initially self‐quenched SRB upon fusion between the vesicles and cells (1–2 × 10^6^). All content‐mixing experiments were performed in the presence of 5 U Thr or 10 U 3C proteases. When specified, the spike‐cell (or uninfected‐cell) or ACE2‐vesicle (or blank‐vesicle) were pre‐cultured with 1 µm Biotin‐PE, 1 µm cholesterol linked DNA strand, or 1 µm PE linked PEG5000. For the ensemble cell–vesicle content mixing shown in Figure 2c,d; Figure S10c and S10d (Supporting Information), spike‐cell (1–2 × 10^6^) with 24 h culture or the spike variant‐cell (1–2 × 10^6^) with 48 h culture were mixed with ACE2‐vesicle in the presence of 5 U Thr protease. For the ensemble cell–vesicle content mixing shown in Figure 2h,i; Figure S10h and S10i (Supporting Information), ACE2‐vesicle at different molar ratios of ACE2 to lipids were mixed with spike‐cell (1–2 × 10^6^) in the presence of 5 U Thr protease. For the ensemble cell–vesicle content mixing in Figure 5c,d, spike‐cell (1–2 × 10^6^) and blank‐vesicle were pre‐cultured with different pairs of cholesterol‐linked complementary DNA strands. For the ensemble cell–vesicle content mixing in Figure 5e,f, spike‐cell (1–2 × 10^6^) and blank‐vesicle were pre‐cultured with different concentrations of cholesterol‐linked DNA strands A2 and D3, respectively. Redundant DNA strands were removed by three rounds of washing with 500 µL PBS (for spike‐cell) or by size exclusion chromatography using the Sepharose CL‐4B column (for blank‐vesicle). For the ensemble cell–vesicle content‐mixing assay shown in Figure S5b and S5c, ACE2‐cell (1–2×10^6^) with 24–48 h culture in logarithmic growth phase was mixed with spike^1268^‐vesicle in the presence of 5 U Thr protease or 10 U 3C proteases. For the ensemble cell–vesicle content‐mixing assay shown in Figure S2b, S2c, S2g, and S2h (Supporting Information), 30 nm furin (Abcam) or 30 nm TMPRSS2 (Abcam) protease was added into the mixture of ACE2‐vesicle and spike^WT^‐cell in vesicle buffer. The fusion probability was calculated by dividing the real‐time intensity of SRB in the content‐mixing assay divided by the intensity of SRB obtained by adding 0.1% Triton X‐100 (Sigma). All cell–vesicle fusion experiments were performed using Duetta^TM^ (Horiba) or Cary Eclipse Fluorescence Spectrophotometer (Agilent) at 25 °C.

### Flow Cytometry Measurement

Briefly, 1×10^6^ HEK293T cells that were transfected with plasmid encoding specified protein were collected using 0.25 mg mL^−1^ trypsin (Beyotime), followed by two rounds of centrifugation at 3000 rpm for 3 min at 4 °C and washing with 500 µL PBS. Then 1 × 10^6^ cells were incubated with 5 µg mL^−1^ anti‐S2 primary antibody (1A9) (GeneTex) for 30 min at 4 °C, followed by three rounds of washing with 500 µL PBS to remove unbound primary antibody. Next, cells were incubated with 1.88 µg mL^−1^ Alexa Fluor 488‐labeled goat anti‐mouse IgG (H+L) secondary antibody (ZSGB‐BIO) or 500 ng mL^−1^ APC‐labeled goat anti‐mouse IgG secondary antibody (Biolegend) for 30 min at 4 °C. All samples were washed with 500 µL PBS for three times to remove the nonspecific binding antibodies and were monitored by FCM. For determining the expression level of ACE2, 1×10^6^ cells were incubated with 1.07 µg mL^−1^ rabbit polyclonal anti‐ACE2 primary antibody (SinoBiological), followed by incubating with 1.88 µg mL^−1^ Alexa Fluor 488‐labeled goat anti‐rabbit IgG (H+L) secondary antibody, the others steps were the same as the aforementioned ones. The NovoExpress software (ACEA Biosciences) was used to analyze the data.

### Dot Blot Assay

Samples containing 60 ng of DNA were immobilized onto a nylon membrane by exposure to UV light (1.5 J) of UV for 20 min). After that, the membrane was blocked with 5 wt% skim milk in PBST (135 mm NaCl, 4.7 mm KCl, 10 mm Na_2_HPO_4_, 2 mm NaH_2_PO_4_, 0.02% Tween20, pH 7.4) at room temperature for 1 h. Next, the membrane was incubated with 1 µg mL^−1^ anti‐DNA primary antibody (Progen) in PBST containing 5 wt.% skim milk overnight at 4 °C. After washing for three times with 5 mL PBST, the membrane was incubated with 80 ng mL^−1^ HRP‐conjugated secondary antibody (ZSGB‐BIO) at 37 °C for 2 h. Finally, after washing for another three times with 5 mL PBST, DNA on the membrane was exposed by using the HRP substrate luminol reagent with a volume ratio of 1:1.

### Surface Plasmon Resonance

All SPR experiments were performed at room temperature using a CM5 sensor chip (GE Healthcare) on the Biacore 8K system (GE Healthcare). To determine the binding affinity of RBD^WT^ to ACE2 or its variants, RBD^WT^ was immobilized on a CM5 sensor chip using the Amine Coupling Kit (GE Healthcare). Gradient concentrations of ACE2, or its variants were added to the buffer (25 mm HEPES, 150 mm NaCl, 0.05% Tween‐20, pH 7.5) at a rate of 30 µL min^−1^. To determine the binding affinity of ACE2 to the RBD or its variant, ACE2 was immobilized on the CM5 sensor chip using the Amine Coupling Kit. Gradient concentrations of RBD, or its variants were added to the buffer (25 mm HEPES, 150 mm NaCl, 0.05–0.07% Tween‐20, pH 7.5). After each cycle, the chip was regenerated using 10 mm glycine (pH 1.5) (GE Healthcare) for 60 s. The kinetic data were further analyzed using the Biacore Insight Evaluation Software for the dissociation constant (Kd) with a 1:1 (Langmuir) binding model for the slow‐on/slow‐off data. For each test, at least independent kinetic assays were performed, and the calculated kinetic parameters were summarized.

### Confocal Microscopy

HEK293T cells were seeded into 6‐well plate and transfected with 6 µg plasmid encoding spike^1268^ using the Lipo8000. After transfection for 48 h, spike^1268^‐cell were collected and pre‐incubated with 1 µm biotin‐PE or 1 µm cholesterol linked DNA‐D for 30 min, while ACE2‐cell or uninfected‐cell were pre‐incubated with 1 µm biotin‐PE or 1 µm cholesterol linked DNA‐A for 30 min. Subsequently, spike‐cell (2×10^5^) and ACE2‐cell (1×10^5^), or uninfected‐cell (1×10^5^) were seeded into 24‐well plates (BIOFIL) and co‐cultured in DMEM containing 10% FBS for 48 h, followed by fixation with 4% paraformaldehyde (Servicebio) for 15 min at room temperature. After fixation, cells were incubated with 1 µg mL^−1^ anti‐S2 primary antibody (1A9) in PBS for 2 h at 4 °C, followed by three rounds of washing with 1 mL PBS per well to remove excess antibodies. Then, the cells were incubated with 500 ng mL^−1^ APC labeled goat anti‐mouse secondary antibody for 1 h at 4 °C, followed by three rounds of washing with 1 mL PBS to remove nonspecific adsorption of secondary antibody. Cell membranes were stained with phalloidin‐fluorescein isothiocyanate (Beyotime) at 1:200 dilution. After three rounds of washing with 1 mL PBS, the nuclei were stained with 5 mg mL^−1^ DAPI (Beyotime). The slides were mounted onto a Confocal Fluorescence Imaging Microscope (ZEISS LSM880) using an Antifade Mounting Medium (Beyotime). Images were acquired at 63× oil magnification and analyzed using ZEN.

### Cell–Cell Fusion Assay

To prepare spike‐EGFP‐cell, HEK293T cells were co‐transfected with plasmids encoding EGFP and spike or its variants. To prepare ACE2‐cell, HEK293T cells were transfected with plasmids encoding ACE2. When specified, spike‐EGFP‐cell, ACE2‐cell and uninfected‐cell groups were pre‐cultured with 1 µm Biotin‐PE, 1 µm cholesterol linked DNA strands, or 1 µm PE linked PEG5000. Spike‐cell (4×10^4^) and ACE2‐cell (2×10^4^), or uninfected‐cell (2×10^4^) were mixed in 96‐well plates (BIOFIL) and co‐cultured in DMEM containing 10% FBS for 15 min. During the 48‐h incubation with 5 U Thr, the wells were scanned for every 2–3 h and analyzed using IncuCyte S3 at 20× magnification. At least three wells were randomly selected for each condition to analyze the area of the fused and unfused cells.

### Statistical Analysis

In general, Novoexpress, ZEN, ImageJ, Prism and Adobe Illustrator software were used to analyze the data. The *P* values were determined using two‐way analysis of variance (ANOVA) as indicated in each figure legend. *P* values < 0.05 were considered significant, n.s., means no significance.

## Conflict of Interest

The authors declare no conflict of interest.

## Author Contributions

M.W. and W.L. contributed equally to this work. M.W., G.L., and Y.L. designed the experiments. M.W., W.L., Y.X., L.C., and W. S performed the cell–vesicle fusion experiments. M.W., W.L., S.L., Y.D., Z.C and F.Y. purified proteins, M.W. and W.L. performed SPR experiments. M.W. and W.L. performed the cell–cell fusion experiments. K.L. performed the TEM experiments. M.W. and W.L. analyzed these data. M.W., J.F., and Y.L. drafted the manuscript.

## Supporting information

Supporting Information

## Data Availability

The data that support the findings of this study are available from the corresponding author upon reasonable request.
