# Supplementary material for: A Suitable Membrane Distance Regulated by the RBD_ACE2 Interaction is Critical for SARS‐CoV‐2 Spike‐Mediated Viral Invasion
Source: Adv Sci (Weinh). 2023 Aug 17;10(28):2301478. doi: 10.1002/advs.202301478 (PMC10558659; doi:10.1002/advs.202301478)
Supplement: Supplementary file 1 — Supporting Information [file ADVS-10-2301478-s001.pdf]

ADVANCED SCIENCE
Open Access

Supporting Information

for *Adv. Sci.*, DOI 10.1002/advs.202301478

A Suitable Membrane Distance Regulated by the RBD_ACE2 Interaction is Critical for SARS-CoV-2 Spike-Mediated Viral Invasion

*Mengdan Wu, Wei Li, Sheng Lin, Jiaqi Fan, Lele Cui, Yijuan Xiang, Kaiyu Li, Linwei Tang, Yanping Duan, Zimin Chen, Fanli Yang, Weiwei Shui, Guangwen Lu and Ying Lai**

Supporting Information

A suitable membrane distance regulated by the RBD_ACE2 interaction is critical for SARS-CoV-2 spike-mediated viral invasion

Mengdan Wu[1#], Wei Li[1#], Sheng Lin[2], Jiaqi Fan[1], Lele Cui[1], Yijuan Xiang[1], Kaiyu Li[1], Linwei Tang[1], Yanping Duan[2], Zimin Chen[2], Fanli Yang[2], Weiwei Shui[1], Guangwen Lu[2], Ying Lai[1,*]

[1]National Clinical Research Center for Geriatrics, State Key Laboratory of Biotherapy, West China Hospital, Sichuan University, Chengdu, Sichuan 610041, China.

[2]West China Hospital Emergency Department, State Key Laboratory of Biotherapy, West China Hospital, Sichuan University, Chengdu, Sichuan 610041, China

[#]These authors contribute equally to this work.

[*]Correspondence: ylai@scu.edu.cn.

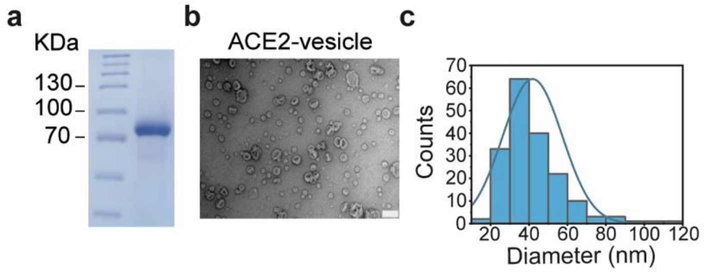

Figure S1. Reconstitution of ACE2-vesicle. a. SDS-PAGE analysis of ACE2-vesicle was shown. **b.** A negative-stain EM image of ACE2-vesicle. Scale bar, 100 nm. **c.** The diameter distribution of ACE2-vesicle.

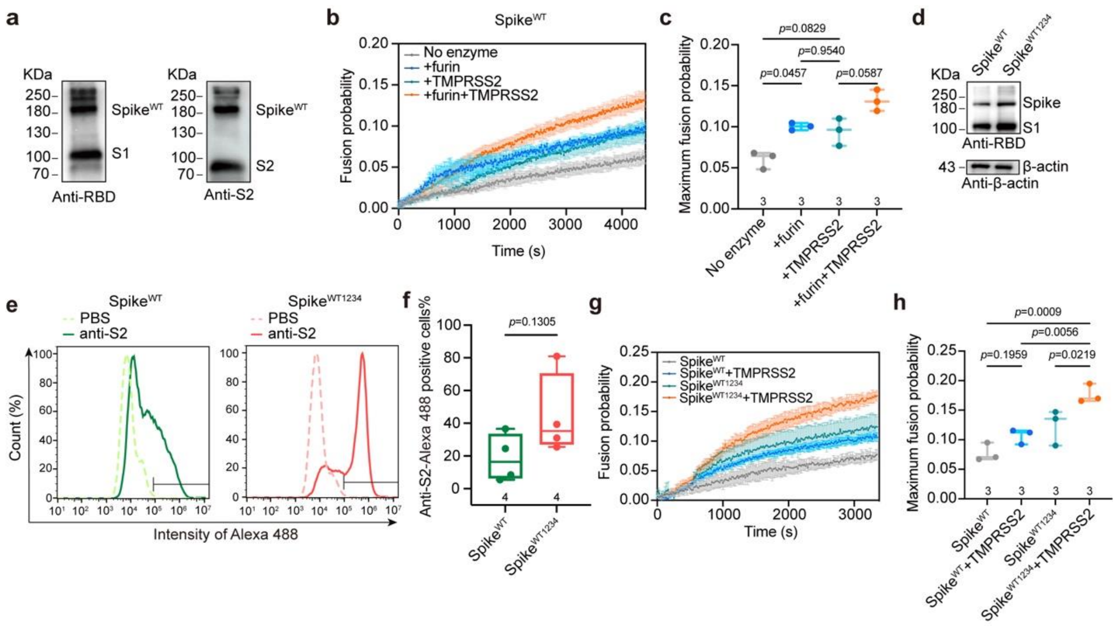

Figure S2. Reconstitution of spike^{WT}-mediated membrane fusion in the cell–vesicle fusion system. a. Endogenous cleavage of spike^{WT} during expression. Western blot analysis of spike^{WT}-cell was performed with the anti-RBD and anti-S2 primary antibodies. **b.** The ensemble content-mixing of the spike^{WT}-cell with ACE2-vesicle. 30 nM furin or 30 nM TMPRSS2 was included when specified. The fluorescence change of SRB for content-mixing was normalized with respect to the fluorescence intensity obtained by adding 0.1% Triton X-100. Traces represent mean ± SEM from multiple repeats of independent experiments. **c.** Box plots and data points show the maximum fusion probability of the spike^{WT}-cell and ACE2-vesicle corresponding to panel b. **d.** Western blot analysis of the expression of spike^{WT}, and spike^{WT1234} via the anti-RBD primary antibody. **e.** One representative data of FCM shows the expression level of spike^{WT}, and spike^{WT1234} on the surface of HEK293T cells. The monoclonal antibody 1A9 was used to target S2, and the expression signal from Alexa 488 labeled secondary antibody was detected. **f.** Box plots and data points show the averaged percentage of the spike-positive cells corresponding to panel e. **g.** The ensemble content-mixing of the spike^{WT}-cell with ACE2-vesicle. 30 nM TMPRSS2 was included when specified. Traces represent mean ± SEM from multiple repeats of independent experiments. **h.** Box plots and data points show the maximum fusion probability of the spike^{WT}-cell and ACE2-vesicle corresponding to panel g. In panels c, f, and h, the repeats (N) of independent experiments were shown above the x-axis, and statistical analysis was performed using two-way ANOVA followed by Tukey's multiple comparisons test.

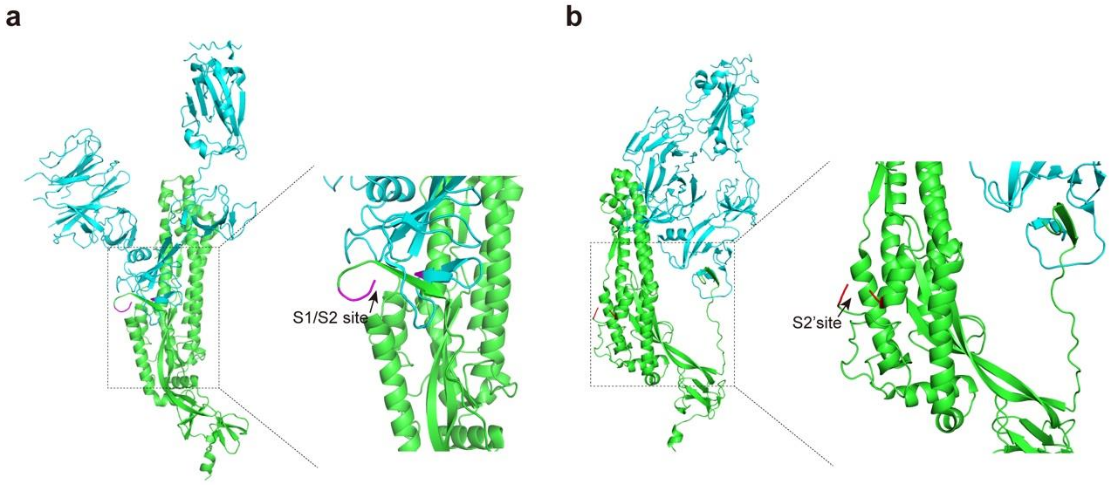

Figure S3. The structure of the S1/S2 and S2’ cleavage sites in spike. a. The S1/S2 site in spike. **b.** The S2’ site in spike. (PDB:6VSB). The S1 and S2 subunits are colored in blue and green, respectively.

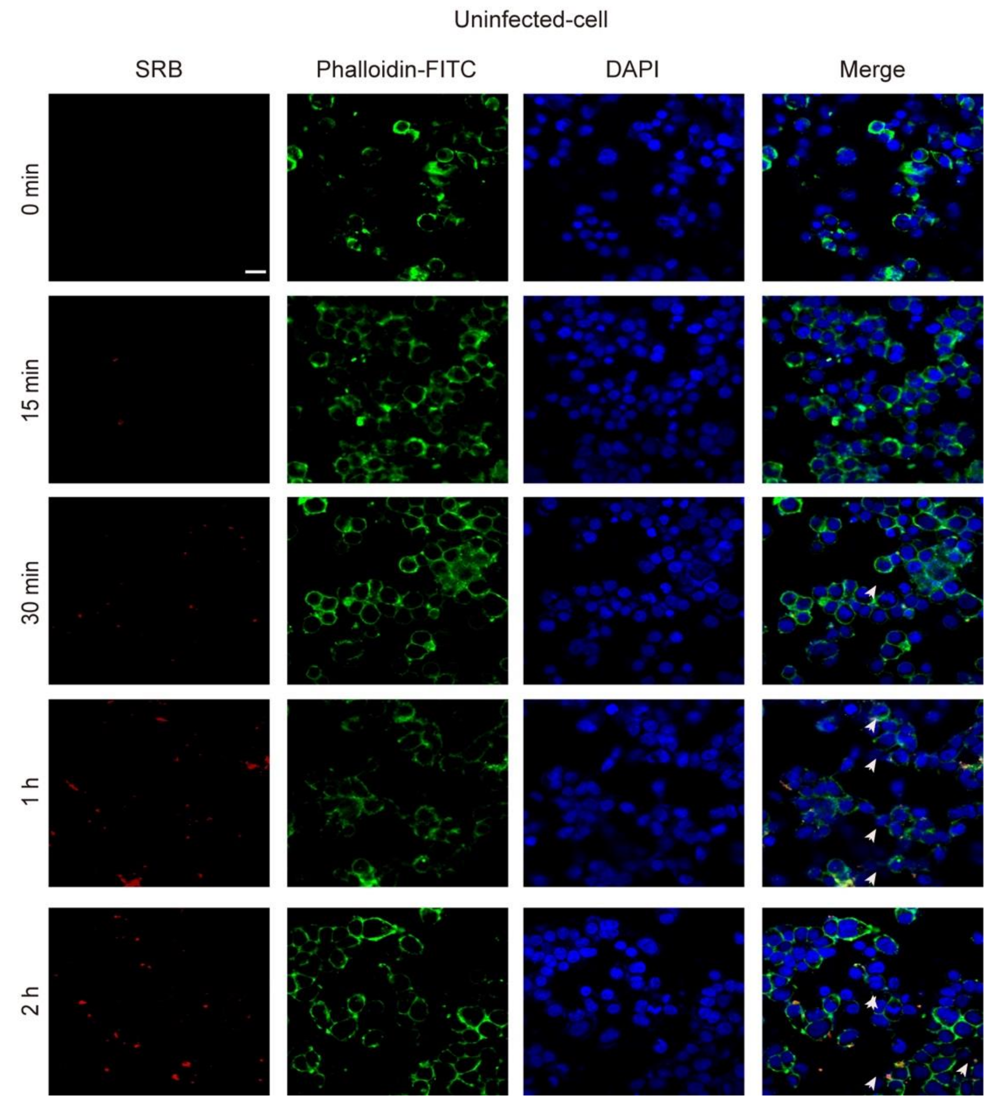

Figure S4. Uptake of the blank-vesicle by uninfected-cell. The uptake of the blank-vesicle by uninfected-cell was observed at different time by laser scanning confocal microscopy. Blank-vesicle containing SRB is shown in red. The cell membrane stained with phalloidin-FITC is shown in green. Nuclear stained with DAPI is shown in blue. Scale bar, 20 µm. White arrows indicate the blank-vesicle uptake by uninfected-cell.

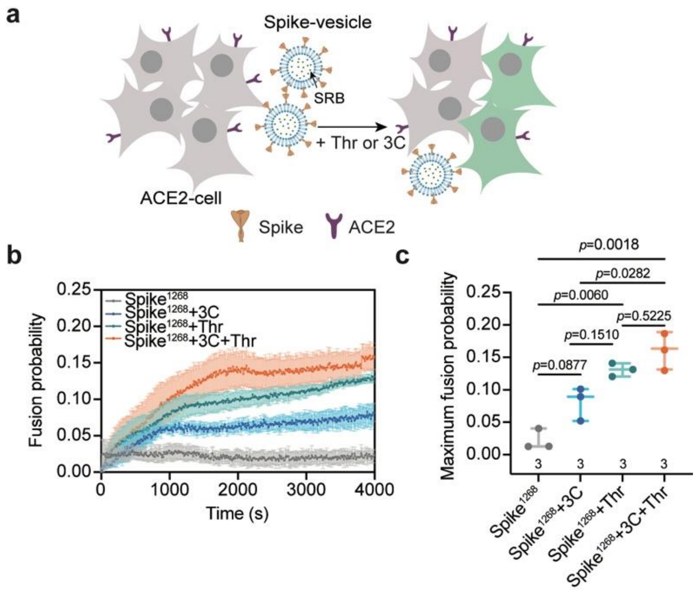

Figure S5. The ensemble content-mixing between the spike^{1268}-vesicle and ACE2-cell. a. Schematic diagram showing the ensemble content-mixing assay between the spike^{1268}-vesicle and ACE2-cell. In the cell–vesicle content-mixing assay, ACE2 was expressed on the surface of HEK293T cell, and purified spike^{1268} was reconstituted onto a group of liposomes. The spike^{1268}-vesicle and ACE2-cell were mixed with 10 U 3C or 5 U Thr protease, and the fluorescence of dequenching signal of SRB was detected. **b**. The ensemble content-mixing of the spike^{1268}-vesicle with ACE2-cell. Traces represent the mean ± SEM from multiple repeats of independent experiments. **c**. Box plots and data points show the maximum fusion probability corresponding to panel b. In panel c, the repeats (N) of independent experiments were shown above the x-axis, and statistical analysis was performed using two-way ANOVA followed by Tukey's multiple comparisons test.

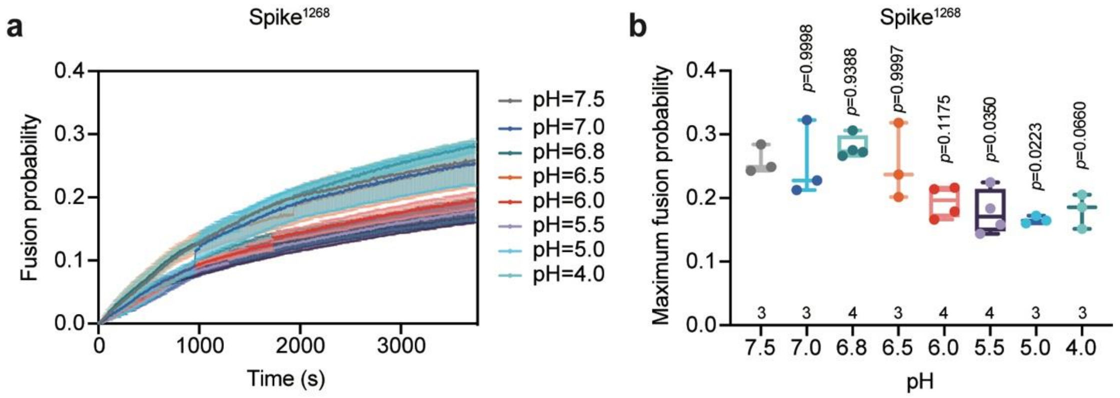

Figure S6. Low pH does not promote spike-mediated membrane fusion. a. The ensemble content-mixing of the spike^{1273}-cell with ACE2-vesicle at different pH in the presence of 5 U Thr. Traces represent mean ± SEM from multiple repeats of independent experiments. **b.** Box plots and data points show the maximum fusion probability corresponding to panel a. The repeats (N) of independent experiments were shown above the x-axis, and statistical analysis was performed using one-way ANOVA followed by Dunnett's multiple comparisons test.

a

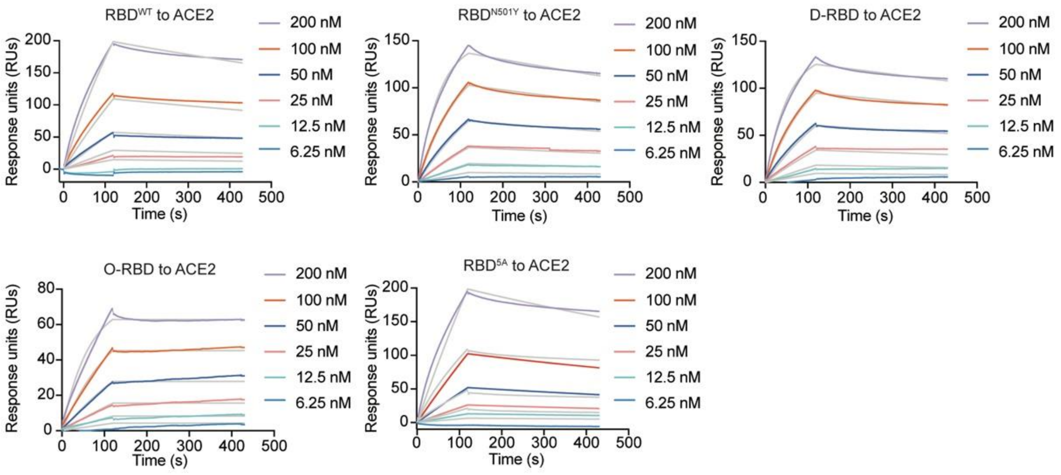

b

Binding pair	ka(1/Ms)	kd(1/s)	Rmax(RU)	KD(M)	Average KD(M)
RBD^{WT} to ACE2	2.91×10^{4}	2.46×10^{-3}	52.7	8.44×10^{-8}	7.21×10^{-8}
	3.93×10^{4}	3.21×10^{-3}	33.5	8.17×10^{-8}	
	4.87×10^{4}	3.72×10^{-3}	93.6	7.63×10^{-8}	
	1.57×10^{4}	7.19×10^{-4}	669.6	4.59×10^{-8}	
RBD^{N501Y} to ACE2	6.09×10^{4}	1.87×10^{-3}	271.5	3.07×10^{-8}	2.82×10^{-8}
	8.34×10^{4}	1.77×10^{-3}	209.4	2.12×10^{-8}	
	1.61×10^{5}	5.10×10^{-3}	293.4	3.18×10^{-8}	
	2.28×10^{4}	6.62×10^{-4}	543.2	2.90×10^{-8}	
D-RBD to ACE2	1.51×10^{5}	5.33×10^{-3}	58.8	3.54×10^{-8}	2.96×10^{-8}
	1.21×10^{5}	3.64×10^{-3}	55.6	3.00×10^{-8}	
	1.10×10^{5}	2.90×10^{-3}	34.5	2.63×10^{-8}	
	2.95×10^{4}	7.86×10^{-4}	406	2.66×10^{-8}	
O-RBD to ACE2	7.11×10^{5}	5.80×10^{-3}	44.9	8.16×10^{-9}	1.91×10^{-8}
	2.27×10^{5}	7.57×10^{-3}	17.2	3.33×10^{-8}	
	2.88×10^{4}	8.62×10^{-4}	378.2	2.87×10^{-8}	
	1.01×10^{5}	6.35×10^{-4}	155	6.28×10^{-9}	
RBD^{5A} to ACE2	1.92×10^{4}	3.79×10^{-3}	155	1.98×10^{-7}	1.85×10^{-7}
	1.36×10^{4}	3.24×10^{-3}	311.1	2.39×10^{-7}	
	2.08×10^{4}	4.31×10^{-3}	145.6	2.07×10^{-7}	
	1.07×10^{4}	1.06×10^{-3}	1259.9	9.89×10^{-8}	

Figure S7. Analysis of the binding kinetics of ACE2 with RBD^{WT} or its mutants. **a.** The real-time binding kinetics of RBD^{WT} or its mutants to ACE2 were characterized by SPR. In the experiment, RBD^{WT}, or its mutants at various concentrations were flowed into the CM5 sensor chip that was coated with ACE2. **b.** Four independent experiments are conducted and the recorded profiles are shown. The calculated kinetic parameters are summarized.

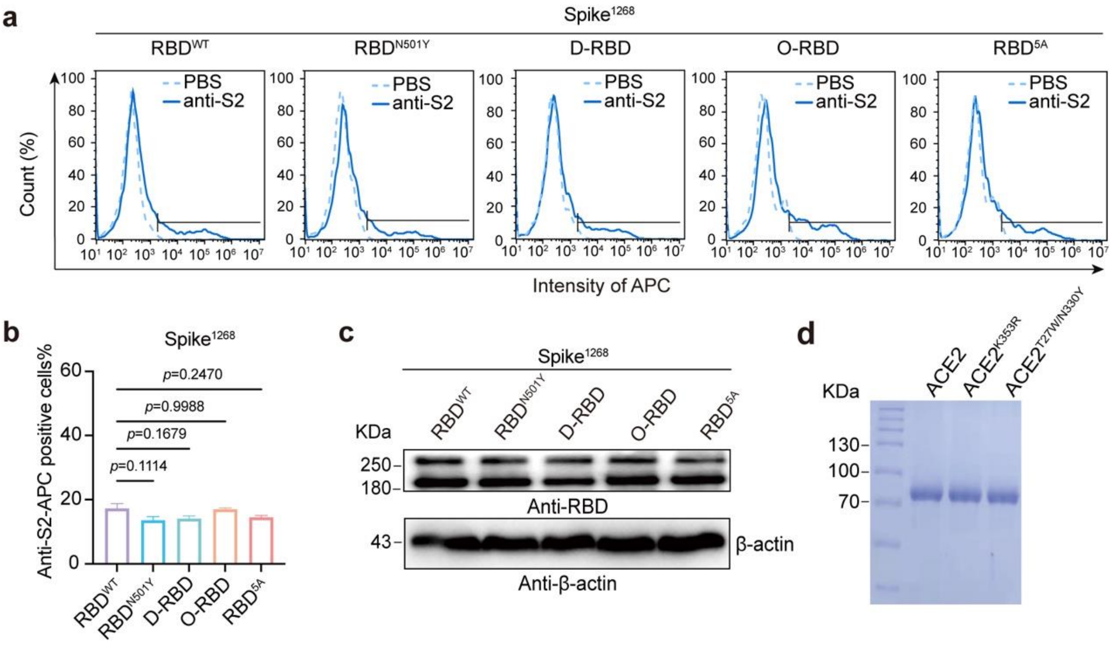

Figure S8. The protein level of spike^{1268}, ACE2 and their mutants on cell surface, or on vesicle. a. One representative data of FCM shows the expression of spike^{1268} and its variants on cell surface. The population of spike^{1268}-positive cells is indicated by horizontal bars. **b.** The averaged percentage of spike^{1268}-positive cells corresponding to panel a. Error bars that represent standard deviation are from 2 independent measurements with 2 different preparations. **c.** Western blot analysis of the expression of spike^{1268} and its variants via the anti-RBD primary antibody. **d.** SDS-PAGE gel analysis of ACE2 and its variants reconstituted vesicles.

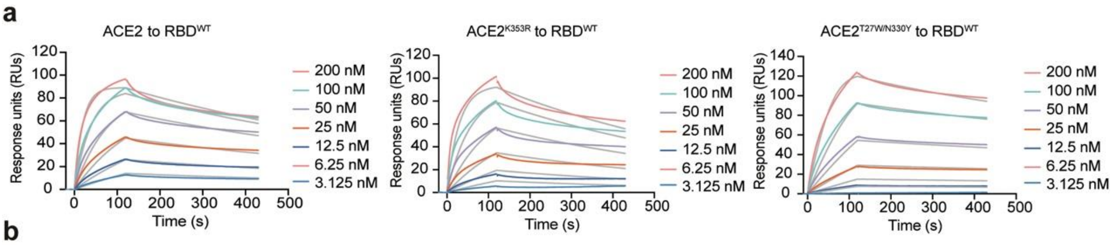

b

Binding pair	ka(1/Ms)	kd(1/s)	Rmax(RU)	KD(M)	Average KD(M)
ACE2 to RBD^{WT}	2.28×10^{4}	1.80×10^{-3}	36.9	7.91×10^{-8}	7.57×10^{-8}
	1.84×10^{4}	1.68×10^{-3}	111.8	9.15×10^{-8}	
	4.46×10^{4}	2.51×10^{-3}	40.5	5.64×10^{-8}	
$ACE2^{K353R}$ to RBD^{WT}	1.50×10^{4}	1.85×10^{-3}	117.4	1.24×10^{-7}	1.84×10^{-7}
	8.97×10^{3}	1.86×10^{-3}	195.4	2.08×10^{-7}	
	8.97×10^{3}	1.97×10^{-3}	196.9	2.19×10^{-7}	
$ACE2^{T27W/N330Y}$ to RBD^{WT}	5.61×10^{4}	1.01×10^{-3}	172.3	1.80×10^{-8}	1.93×10^{-8}
	5.20×10^{4}	1.11×10^{-3}	167.7	2.13×10^{-8}	
	6.58×10^{4}	1.22×10^{-3}	185.4	1.86×10^{-8}	

Figure S9. Analysis of the binding kinetics of RBD^{WT} with ACE2, or its mutants. a. The real-time binding kinetics of RBD^{WT} to ACE2 or its mutants were characterized by SPR. ACE2, or its variants at various concentrations were flowed into the CM5 sensor chip that was coated with RBD^{WT}. **b.** Three independent experiments are conducted and the recorded profiles are shown. The calculated kinetic parameters are summarized.

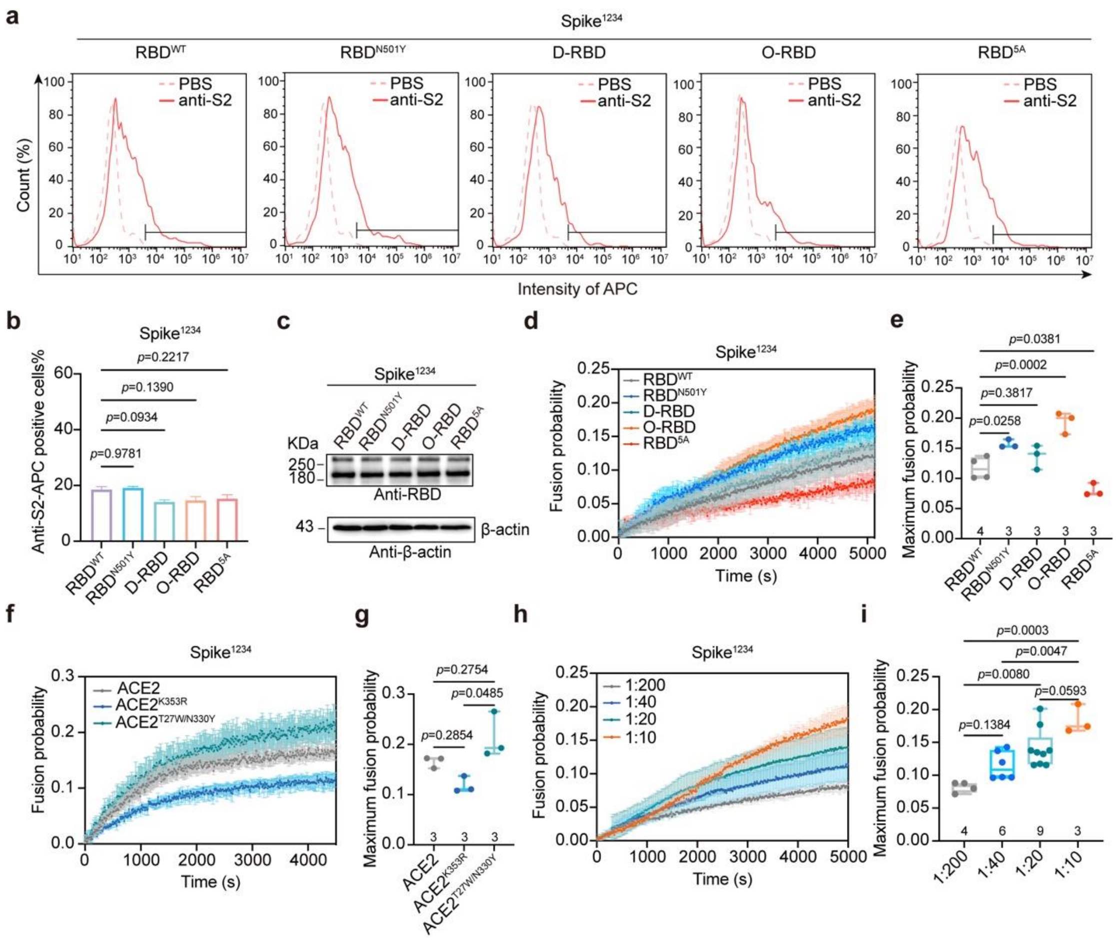

Figure S10. Binding affinity between RBD to ACE2 is positively correlated with spike^{1234}-mediated membrane fusion. a. One representative data of FCM shows the expression of spike^{1234} and its variants on the cell surface. The population of spike^{1234}-positive cells is indicated by horizontal bars. **b.** The averaged percentage of spike^{1234}-positive cells corresponding to panel a. Error bars that represent standard deviation were from 2 independent measurements with 2 different preparations. **c.** Expression of spike^{1234} and its variants were detected by western blot. **d.** The ensemble content-mixing assay of the spike^{1234} or its mutants-expressing cells with ACE2-vesicle in the presence of 5 U Thr protease. Traces represent mean ± SEM from multiple repeats of independent experiments. **e.** Box plots and data points show the maximum fusion probability corresponding to panel d. **f.** The ensemble content-mixing of the spike^{1234}-cell with vesicles reconstituted with ACE2 or its mutants in the presence of 5 U Thr protease. Traces represent mean ± SEM from multiple repeats of independent experiments. **g.** Box plots and data points show the maximum fusion probability corresponding to panel f. **h.** The ensemble content-mixing of the spike^{1234}-cell with ACE2-vesicle at different molar ratios of ACE2 : lipids in the presence of 5 U Thr protease. Traces represent mean ± SEM from multiple repeats of independent experiments. **i.** Box plots and data points show the maximum fusion probability corresponding to panel h. In panels e, g, and i, the repeats (N) of independent experiments were shown above the x-axis, and statistical

analysis was performed using two-way ANOVA followed by Tukey's multiple comparisons test.

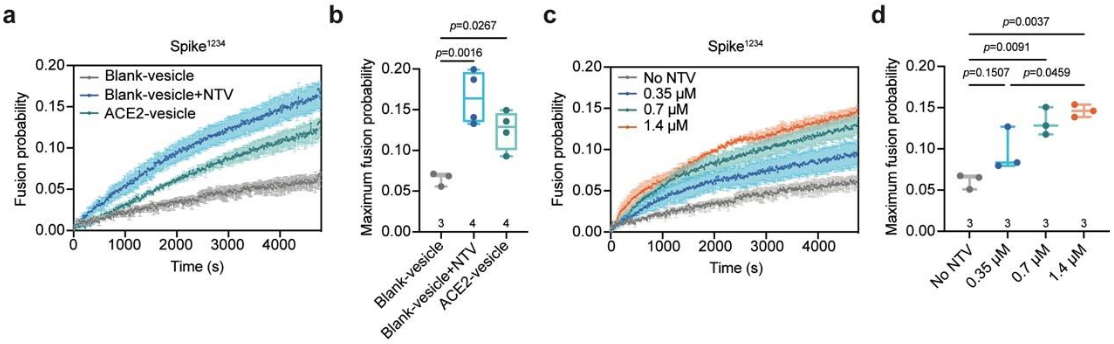

Figure S11. Crosslinking membranes by the NTV_biotin interaction drives spike^{1234}-mediated membrane fusion. a. The cell–vesicle content-mixing of the spike^{1234}-cell with ACE2-vesicle or blank-vesicle in the presence of 5 U Thr protease. 0.7 μM NTV was included when specified. Traces represent mean ± SEM from multiple repeats of independent experiments. **b.** Box plots and data points show the maximum fusion probability corresponding to panel a. **c.** The ensemble cell–vesicle content-mixing of the spike^{1234}-cell with blank-vesicle at specified concentrations of NTV in the presence of 5 U Thr protease. Traces represent mean ± SEM from multiple repeats of independent experiments. **d.** Box plots and data points show the maximum fusion probability corresponding to panel c. In panels b and d, the repeats (N) of independent experiments were shown above the x-axis, and statistical analysis was performed using two-way ANOVA followed by Tukey's multiple comparisons test.

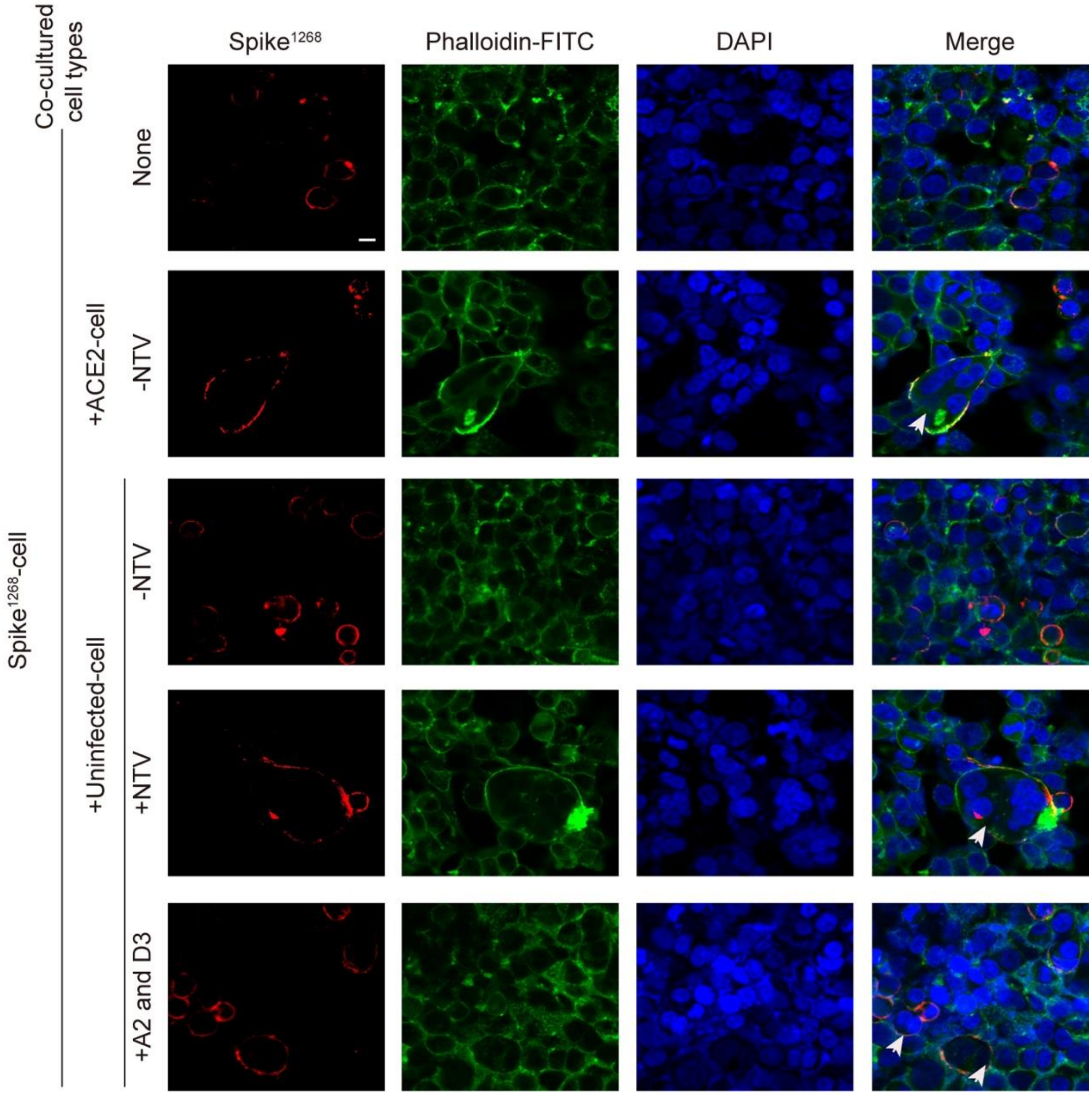

Figure S12. Examination of the spike^{1268} induced syncytia formation by confocal microscopy. Cell–cell fusion at specified conditions was shown. The spike^{1268} was detected via staining with the 1A9 primary antibody and followed by the APC labeled secondary antibody as shown in red. The cell membrane stained with phalloidin-FITC is shown in green. Nuclear stained with DAPI is shown in blue. The syncytia formation is indicated by white arrows. Scale bar, 10 μm.

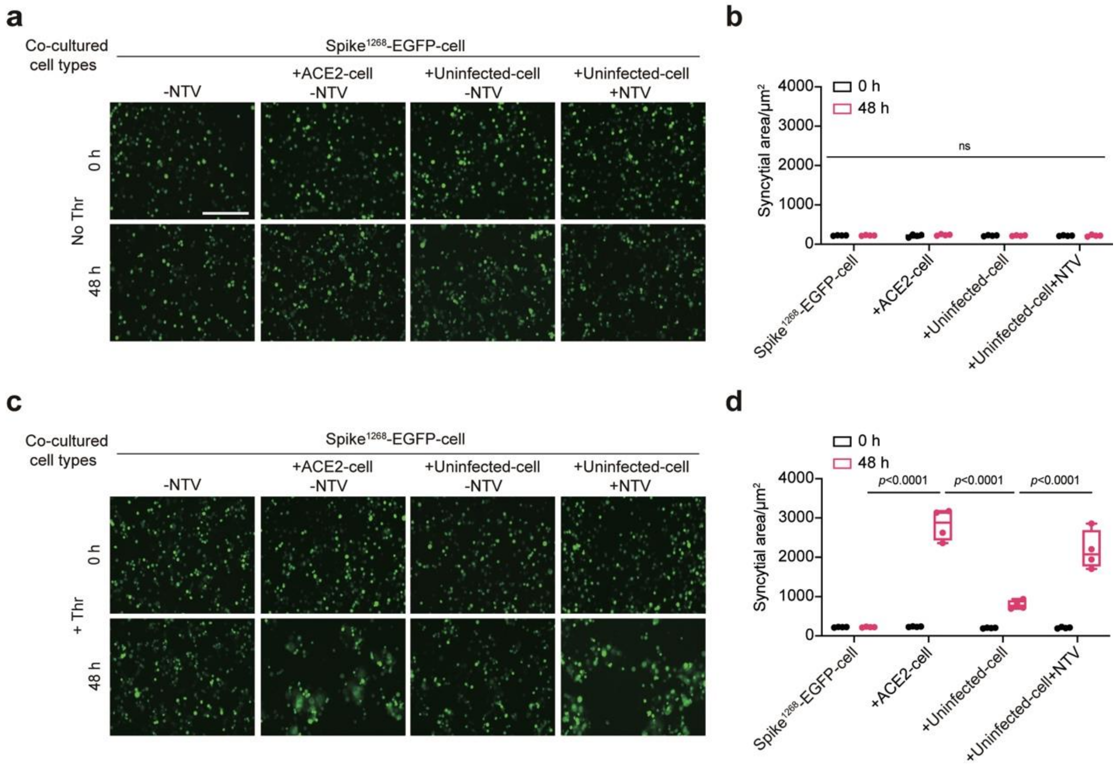

Figure S13. spike^{1234}-mediates cell–cell fusion by crosslinking membrane via the NTV_biotin interaction. a. Syncytia formation induced by spike^{1234}-mediated cell–cell fusion in the absence of Thr. After spike^{1234}-EGFP-cell, ACE2-cell, and uninfected-cell were treated with biotin-PE, spike^{1234}-EGFP-cell and ACE2-cell (or uninfected-cell) were co-cultured at a 2:1 ratio without Thr protease. Scale bar, 200 μm. **b.** Box plots and data points show the area of the syncytia in cell–cell fusion corresponding to panel a. **c.** Syncytia formation induced by spike^{1234}-mediated cell–cell fusion in the presence of 5 U Thr. After spike^{1234}-EGFP-cell, ACE2-cell, and uninfected-cell were treated with biotin-PE, spike^{1234}-EGFP-cell and ACE2-cell (or uninfected-cell) were co-cultured at a 2:1 ratio with 5 U Thr protease. Scale bar, 200 μm. **d.** Box plots and data points show the area of the syncytia in cell–cell fusion corresponding to panel c. The area of the syncytia was calculated based on the fused cells with weak fluorescence intensity of EGFP and were at least twice as large as the unfused cells. At least five fields were randomly selected in each well of experiment. Statistical analysis was performed using two-way ANOVA followed by Tukey's multiple comparisons test.

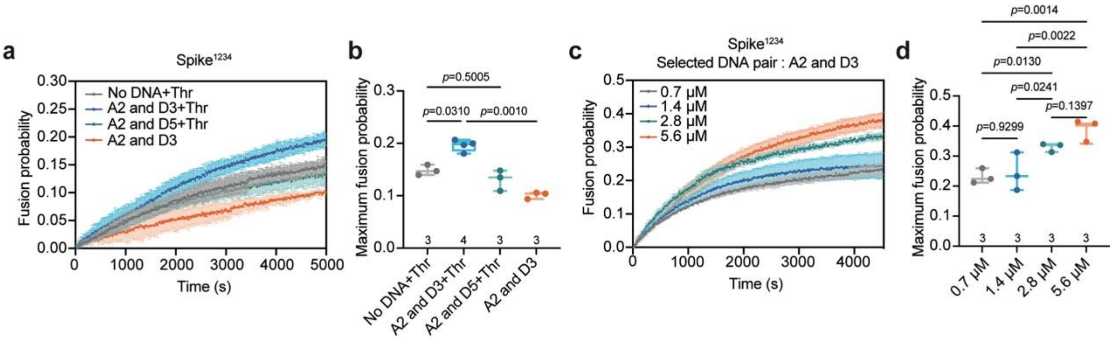

Figure S14. Spike[1234] fuses two membranes that are crosslinked by complementary DNA strands. a. The ensemble cell–vesicle content-mixing of the spike[1234]-cell with blank-vesicle by using different lengths of DNA strands in the presence of 5 U Thr protease. Traces represent mean ± SEM from multiple repeats of independent experiments. **b.** Box plots and data points show the maximum fusion probability corresponding to panel a. **c.** The ensemble cell–vesicle content-mixing assay of the spike[1234]-cell with blank-vesicle at specified concentration of DNA strands (A2 and D3). Traces represent mean ± SEM from multiple repeats of independent experiments. **d.** Box plots and data points show the maximum fusion probability corresponding to panel c. In panels b and d, the repeats (N) of independent experiments were shown above the x-axis, and statistical analysis was performed using two-way ANOVA followed by Tukey’s multiple comparisons test.

Table S1. Information of all cholesterol-linked DNA strands.

DNA name	Length	DNA sequences (5'-3')
DNA-D1	12 bp	Cholesterol-TCG ACA CGG AAA
DNA-D2	24 bp	Cholesterol-TCG ACA CGG AAA TGT TGA ATA CTA
DNA-D3	36 bp	Cholesterol-C×12 TCG ACA CGG AAA TGT TGA ATA CTA
DNA-D4	66 bp	Cholesterol-C×42 TCG ACA CGG AAA TGT TGA ATA CTA
DNA-D5	96 bp	Cholesterol-C×72 TCG ACA CGG AAA TGT TGA ATA CTA
DNA-D6	129 bp	Cholesterol-C×105 TCG ACA CGG AAA TGT TGA ATA CTA
DNA-A1	12 bp	Cholesterol-TTT CCG TGT CGA
DNA-A2	24 bp	Cholesterol-TAG TAT TCA ACA TTT CCG TGT CGA
Short DNA-D	6 bp	Cholesterol-TCG ACA
Short DNA-A	6 bp	Cholesterol-TGT CGA
Long DNA-D	129 bp	Cholesterol-A×105 TCG ACA CGG AAA TGT TGA ATA CTA
Long DNA-A	129 bp	Cholesterol-TAG TAT TCA ACA TTT CCG TGT CGA T×105
